# Water-Content Dependence of ZnO Crystallite Size in Single-Step Microwave Solvothermal Co-Synthesis of ZnO/Ag Nanocomposites

**DOI:** 10.3390/ma19214520

**Published:** 2026-10-27

**Authors:** Jacek Wojnarowicz, Urszula Szałaj, Svitlana Stelmakh

**Affiliations:** Laboratory of Nanostructures, Institute of High Pressure Physics, Polish Academy of Sciences, Sokołowska 29/37, 01-142 Warsaw, Poland; u.szalaj@labnano.pl (U.S.); svrit@unipress.waw.pl (S.S.)

**Keywords:** microwave solvothermal synthesis (MSS), ZnO/Ag nanocomposites, metallic silver (Ag^0^), ZnO crystallite size, precursor water content

## Abstract

In microwave solvothermal synthesis (MSS), ZnO particle size increases with precursor water content. This study tested whether that dependence changes when metallic silver forms in the same step. ZnO/Ag nanocomposites with nominally 2 mol% Ag were obtained by single-step MSS from zinc and silver acetates in ethylene glycol, without a separately added reducing agent, at measured precursor water contents of 1.47–3.97 wt%. XRD detected wurtzite ZnO and metallic Ag^0^ but no crystalline silver oxides. As the water content increased, the apparent ZnO crystallite size grew monotonically from 15 to 25 nm in the a–b basal plane and from 18 to 40 nm along the c axis. The BET specific surface area fell from 50.7 to 29.0 m^2^/g and the equivalent diameter *d*_(BET)_ rose from 22 to 38 nm. Standardless EDS gave similar area-averaged Ag estimates for all samples, and AsB imaging of one sample revealed high-Z features consistent with an Ag-rich phase. An Ag-free ZnO reference series synthesised in the same reactor under identical conditions showed a similar dependence on water content. For three pairs of samples with nearly matched water contents, the series differed in specific surface area and *d*_(BET)_ by at most about 7%, much less than the changes with water content. The separate preparation of the two series and the lack of replicate syntheses limit the ability to attribute the observed differences unambiguously to the presence of silver. Antimicrobial and photocatalytic activity was not investigated.

## 1. Introduction

Increasing antimicrobial resistance, together with the lessons learned from the COVID-19 pandemic, has intensified the search for alternative, unconventional antimicrobial materials capable of acting through multiple mechanisms and remaining effective against drug-resistant strains [1,2]. Zinc oxide–silver nanocomposites are a promising class of such materials. Zinc oxide (ZnO) is one of the most extensively studied II–VI semiconductors. It crystallises in the hexagonal wurtzite structure (P6_3_mc) and combines low cost, wide availability, chemical and thermal stability and limited toxicity, which makes it attractive for photocatalytic, antimicrobial, sensing and optoelectronic applications and for protection against UV radiation [3,4,5]. The photocatalytic and antibacterial activity of ZnO arises, among other factors, from the generation of reactive oxygen species (ROS) and the release of Zn^2+^ ions [5]. Importantly, this activity depends strongly on particle size and on the specific surface area. Smaller ZnO particles provide a larger specific surface area, which can enhance ROS generation and Zn^2+^ release. These effects may increase antimicrobial activity [6,7]. In ZnO/Ag composites, Ag^+^ release from the silver phase provides an additional antimicrobial mechanism [8]. For this reason, control of the size of the ZnO phase is one of the key parameters determining the suitability of these materials for their target antimicrobial and photocatalytic applications. The practical use of pure ZnO is, however, limited by the rapid recombination of photogenerated electron–hole pairs, which lowers the photocatalytic efficiency [4,9]. Its antibacterial activity does not require irradiation, although light can enhance the ROS-mediated contribution [7].

One widely studied strategy for addressing this limitation is to modify ZnO with a noble metal, particularly silver [3,4]. At the Ag/ZnO interface, a Schottky barrier is often proposed to trap photogenerated electrons and suppress electron–hole recombination. Silver may also extend visible-light absorption through localised surface plasmon resonance [3,4]. These effects are exploited in the photocatalytic degradation of organic pollutants such as dyes, pesticides and pharmaceutical residues. These processes are applied in water treatment, where the efficiency is jointly determined by charge-carrier separation at the Ag–ZnO interface and by the accessible surface area of the oxide phase [10,11]. The nature of the junction is not unambiguous: in model systems the Ag/ZnO contact may be ohmic rather than rectifying [12].

In antimicrobial applications, Ag^+^ ions can damage cell membranes, proteins and genetic material, and silver nanoparticles have been tested against both drug-resistant and drug-susceptible bacteria [8]. These mechanisms do not require irradiation; together with ROS generated by ZnO, they may broaden the activity of ZnO/Ag against Gram-positive and Gram-negative bacteria [4,5]. For these reasons, ZnO/Ag nanocomposites are being intensively studied as multifunctional materials, including for biomedical use [3,4,5,13]. Besides biomedical uses, they are being developed as antimicrobial components of coatings and food-packaging films, where particle size and dispersibility affect both the antibacterial activity and the optical, mechanical and barrier properties [14,15].

A distinction should be drawn between three types of Ag–ZnO systems: silver may be incorporated into the wurtzite lattice as a substitutional dopant, coexist with ZnO as a separate crystalline phase of metallic Ag^0^, or form a silver oxide phase (e.g., Ag_2_O or AgO). This distinction, often omitted in the literature, is important because the individual cases correspond to different properties and mechanisms of action [3,4].

ZnO/Ag nanocomposites have been obtained by physical methods, including gas-phase synthesis [9], magnetron co-sputtering of thin films [16], laser ablation [17] and ball milling with hot pressing of Ag-matrix composites [18], as well as by chemical [19,20,21,22,23] and biological [4,5,13] methods, reviewed in [3]. Many routes involve two steps: ZnO formation followed by Ag deposition or photoreduction. Others require external reducing agents, such as NaBH_4_ or hydrazine, which may introduce impurities [3,4]. ZnO/Ag performance also depends on particle size and morphology and on the distribution and interfacial contact of Ag with ZnO, which affect charge transfer and activity [3]. This motivates single-step routes that do not require a separately added reducing agent and that form Ag in situ while retaining control over ZnO size.

Microwave solvothermal synthesis (MSS) is well suited to these requirements. Volumetric, rapid and relatively uniform microwave heating can promote rapid nucleation, leading to small nanoparticles with a narrow size distribution [24,25,26,27]. Ethylene glycol couples strongly to microwave radiation and has a high boiling point (~197 °C). In the polyol process, it serves as a solvent, mild reducing agent and stabilising or shape-directing medium [25,28]. Direct volumetric heating can also shorten the reaction time relative to conventional external heating; whether it lowers the total energy demand depends on the process and the reactor [24,29]. In the polyol system, zinc acetate is converted to ZnO through basic double salts such as Zn_5_(OH)_8_(CH_3_COO)_2_·xH_2_O. Esterification, which produces esters and water, is central to ZnO formation [26]. At MSS temperatures, ethylene glycol can also reduce Ag^+^ ions to metallic Ag^0^ without a separately added reducing agent [28]. One-pot polyol routes have already yielded silver–oxide structures such as Ag@TiO_2_ [28], which makes a one-pot synthesis of a two-phase ZnO/Ag^0^ system possible. Microwave heating has likewise been used for the rapid reduction of Ag^+^ to metallic silver; in microwave-heated ethylene glycol containing poly(vinylpyrrolidone), silver acetate was considerably more reactive than silver nitrate [30].

One-pot preparation of ZnO/Ag in ethylene glycol has already been described under reflux or solvothermal conditions, using either sequential formation of ZnO followed by in situ reduction of silver [31] or simultaneous introduction of the precursors [32]. In these approaches, glycol acts as both the solvent and the reducing agent for the conversion of Ag^+^ to Ag^0^. A surfactant-free, single-step synthesis of M/ZnO nanocomposites (M = Pd, Au, Ag, Cu) in refluxed glycol has also been reported [33]. The single-step method is therefore well established. However, it has not been investigated whether the Ag phase formed in this method changes the dependence of the ZnO crystallite size on the precursor water content.

Previous MSS studies showed that ZnO particle size increased from about 20 to 120 nm with precursor water content [25,26]. This trend was attributed to the role of water in the competing hydrolysis and esterification reactions [26]. For ZnO formed from zinc acetate in 2-propanol, particle size also increased with water concentration, but this was attributed to faster diffusion-limited coarsening [34]. The type of alcohol also affects the phase composition of the product and the size and shape of the ZnO particles [35,36]. In our earlier work, we described Ag-free ZnO and a nanocomposite with a nominal Ag content of 1 mol%, both obtained by MSS in a different microwave reactor at a water content of 1.5 wt%. In the composite, a separate crystalline phase of metallic Ag was detected by XRD [37]. A later study covered six formulations: Ag-free ZnO and ZnO with nominal Ag contents of 0.1 and 1 mol%, each at nominal water contents of 1.5 and 6 wt%. Each formulation was represented by one sample, and the particle sizes were determined from the specific surface area. Two of these samples were those reported in [37], and their characterisation data are identical in both publications (Supplementary Materials to [38], Table S2). That study compared the responses of plants to formulations with different water contents.

The separate phase of metallic Ag^0^ that forms may affect the nucleation and growth of ZnO. We therefore investigated whether, in the presence of this phase, the dependence of ZnO crystallite size on precursor water content is similar to that for Ag-free ZnO. Here, four measured water contents (1.47–3.97 wt%) were examined at a fixed nominal Ag content of 2 mol%. Direction-specific Scherrer analysis of the XRD reflections with correction for instrumental broadening, FW1/5/4/5M analysis and BET were used to assess ZnO size; helium pycnometry and SEM/EDS characterised the composites, and AsB compositional-contrast imaging was performed on one sample. A matched Ag-free ZnO reference series, obtained in the same reactor, from the same reagent batches and under the same process conditions, was characterised by the same methods in order to compare the dependence on water content in the presence and in the absence of Ag.

## 2. Materials and Methods

### 2.1. Reagents

The following reagents were used in the synthesis without additional purification: zinc acetate dihydrate (Zn(CH_3_COO)_2_·2H_2_O, analytical grade, Chempur, Piekary Śląskie, Poland), silver(I) acetate (CH_3_COOAg, Chempur, Piekary Śląskie, Poland), ethylene glycol (EG, ethane-1,2-diol, C_2_H_4_(OH)_2_, pure grade, Chempur, Piekary Śląskie, Poland) and deionised water (H_2_O, specific conductivity <0.1 µS/cm, HLP 20UV, Hydrolab, Straszyn, Poland).

### 2.2. Synthesis of ZnO/Ag Nanocomposites and of the Ag-Free ZnO Reference Series

**Scheme 1:**
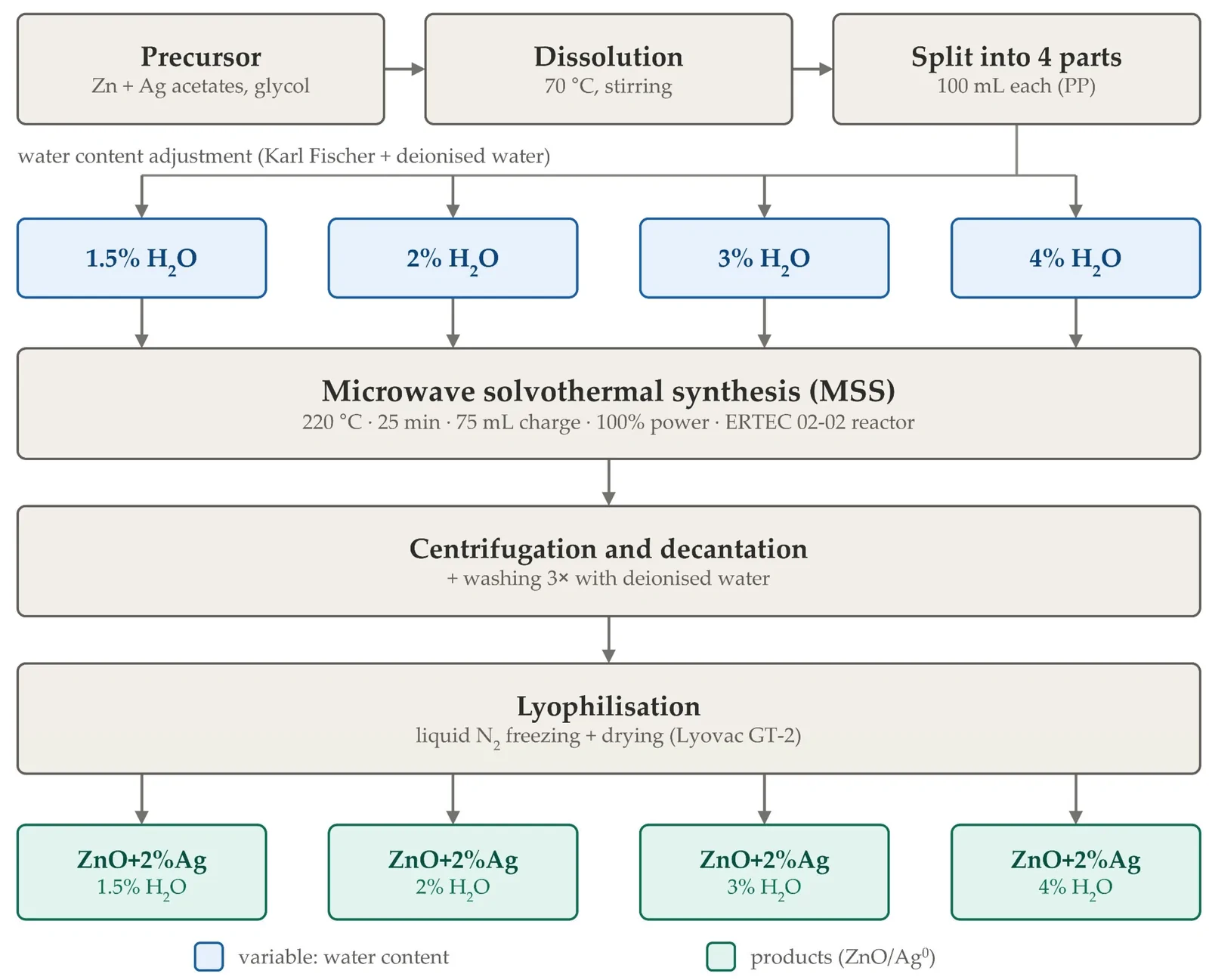
Schematic overview of the preparation of ZnO/Ag nanocomposites by microwave solvothermal synthesis (MSS). The only intentionally varied formulation parameter was the precursor water content (nominal 1.5, 2, 3 and 4 wt%; nominally 2 mol% Ag); the remaining steps were identical for all samples.

ZnO/Ag nanocomposites were obtained by microwave solvothermal synthesis (MSS), following the procedure described in our earlier work [25,26], modified by adding silver(I) acetate to the precursor mixture. Sample preparation is shown schematically in Scheme 1. The amount of silver(I) acetate was calculated using Equation (1).

(1) $$x_{{Ag}^{+}}=\frac{n_{{Ag}^{+}}}{n_{{Ag}^{+}}+n_{{Zn}^{2+}}}\cdot 100\%\;$$

Zn(CH_3_COO)_2_·2H_2_O (26.6650 g) and CH_3_COOAg (0.4138 g) were dissolved in 400 mL of ethylene glycol, giving a nominal Ag/(Ag + Zn) molar fraction of 2%. The solution was heated on a magnetic stirrer with a hot plate (70 °C, 450 rpm, SLR, SI Analytics, Mainz, Germany). After the acetates had completely dissolved, the solution was divided into four equal 100 mL portions, each in a tightly sealed polypropylene container. Once room temperature had been reached, the initial water content of the precursor was determined by Karl Fischer titration to be 1.06 ± 0.01 wt% (n = 3). Of this amount, about 0.93 wt% originated from zinc acetate dihydrate (4.38 g of water of crystallisation, relative to 400 mL of ethylene glycol with a density of 1.113 g/cm^3^), and the remainder was attributed to residual water in the ethylene glycol, which was not determined separately. The calculated amounts of deionised water were then added to obtain the target (nominal) water content of 1.5, 2, 3 and 4 wt%. After the water had been added and the precursors homogenised, the final water content of each precursor was determined in triplicate; these values are the ones reported in Table 1. The synthesis was carried out in a microwave reactor (model 02-02, 600 W, 2.45 GHz, ERTEC, Wrocław, Poland) under conditions identical for all samples. A 75 mL charge taken from each portion was heated at 220 °C for 25 min at the reactor’s nominal maximum power of 600 W. The reaction chamber was then cooled for 20 min. After synthesis, the suspensions were centrifuged (MPW-350, MPW Med Instruments, Warsaw, Poland) and decanted, and the resulting pellets were washed three times with deionised water. The moist pellets were redispersed in water, frozen with liquid nitrogen (Messer Polska, Chorzów, Poland) and dried in a freeze-dryer (Lyovac GT-2, SRK Systemtechnik, Riedstadt, Germany). Freeze-drying was used to limit additional agglomeration and aggregation during drying. The four samples finally obtained were named: ZnO+2%Ag-1.5%H_2_O, ZnO+2%Ag-2%H_2_O, ZnO+2%Ag-3%H_2_O and ZnO+2%Ag-4%H_2_O.

In these sample codes the silver content is the nominal value in mol% of the metals and the water content is the nominal precursor water content in wt%.

For comparison, an Ag-free ZnO reference series was prepared. It was obtained in the same microwave reactor (ERTEC 02-02) under identical process conditions (220 °C, 25 min, nominal power 600 W, 75 mL charge) ten days before the ZnO/Ag series, from the same batches of zinc acetate dihydrate and ethylene glycol and at the same zinc concentration (0.304 mol per litre of ethylene glycol), with silver(I) acetate omitted. The precursor was prepared in a separate dissolution process. Its initial water content, determined by Karl Fischer titration, was 0.98 ± 0.02 wt% (n = 3), of which about 0.93 wt% also originated from zinc acetate dihydrate. The precursor was divided into equal portions. The water content of each portion was then adjusted to the nominal values of 1, 2, 3 and 4 wt% and determined by Karl Fischer titration, as described in Section 2.3. Centrifugation, washing, freeze-drying and all characterisation steps were the same as for the ZnO/Ag series. The samples were designated ZnO-1%H_2_O, ZnO-2%H_2_O, ZnO-3%H_2_O and ZnO-4%H_2_O.

The reference series is matched to the ZnO/Ag series in terms of the reactor, process parameters, reagent batches, zinc concentration and analytical procedures. It differs from it in the omission of silver(I) acetate, the separate preparation of the precursor and the date of synthesis. In each series, one synthesis was performed for each water content, from a portion of the common precursor of the given series.

### 2.3. Water Content Determination

The water content in the precursors was determined by coulometric Karl Fischer titration in an automatic Cou-Lo AquaMAX KF titrator (GR Scientific, Bedford, UK), using Aquagent Coulometric OIL (anolyte) and Aquagent Coulometric CG (catholyte) reagents (Scharlau, Barcelona, Spain). Liquid samples were taken with a glass syringe (1 mL) and weighed on an analytical balance (WAA 100/C/1, RADWAG, Radom, Poland). The results are compiled in Table 1. Because trace water varies among reagent batches and during storage, the precursor water content was measured for each preparation.

### 2.4. X-Ray Diffraction (XRD)

The phase composition of the samples was analysed by X-ray diffraction (XRD) on an X’Pert PRO diffractometer (Panalytical, Almelo, The Netherlands) with a Cu-anode X-ray tube. The diffraction patterns were recorded at room temperature over the 2θ angle range from 10° to 100° with a step of 0.03°. The measurements were performed using CuKα_1_ radiation (*λ* = 1.54056 Å) with an incident-beam monochromator. The reflection profiles were fitted with a pseudo-Voigt function in the Fityk program (version 0.9.8, Marcin Wojdyr, Warsaw, Poland) [39]. Each reflection was described by a single function. The background was described by a quadratic function for the LaB_6_ standard and by a linear function for all samples. The crystallite size was calculated from the Scherrer equation with K = 1. The (100) reflection gave the crystallite dimension in the direction normal to the (100) planes, lying in the a–b basal plane, and the (002) reflection gave the dimension along the c axis, [001]. At K = 1, the width appearing in the equation is the integral breadth, so the reported sizes are volume-weighted. The integral breadth was taken as the ratio of the area of the fitted peak to its maximum height. The reported size is the volume-weighted mean crystallite thickness, measured perpendicular to the reflecting planes [40]. It was not converted to the true crystallite size, because this would require assumptions about the crystallite shape and size distribution [40]. Nor is it equivalent to the particle diameter, since a particle may contain several crystallites. In the remainder of this work, this quantity is referred to for brevity as the crystallite size. The ZnO sizes obtained using the Scherrer equation and FW1/5/4/5M analysis, and the Ag^0^ sizes obtained using the Scherrer equation, are reported as apparent crystallite sizes in Table 2. The Ag (111) reflection is weak at the silver content used, so the profile fitted to it is markedly less well constrained than those of the ZnO reflections, and the Ag^0^ crystallite sizes reported in Table 2 are therefore indicative only. The resulting values are XRD-derived crystallite sizes and, owing to the well-known limitations of the Scherrer method, do not necessarily correspond to whole-particle diameters [40].

The instrumental broadening was determined on the basis of a LaB_6_ powder standard (LaB6, SRM660, NIST, Gaithersburg, MD, USA), measured on the same diffractometer in the same optical configuration as the samples. The (110), (111) and (200) reflections of LaB_6_ at 2θ = 30.5°, 37.5° and 43.6° were fitted in the same way as the sample reflections. For each reflection of the standard and of the samples, the integral breadth β and the ratio φ = FWHM/β were used to separate the profile into a Lorentzian (Cauchy) component β_L_ and a Gaussian component β_G_ [41]. The instrumental components at the position of each sample reflection were read from straight lines fitted to the three values for LaB_6_ as a function of 2θ (*R*^2^ = 0.9999 for β_L_ and 0.996 for β_G_). All reflections used to calculate the crystallite sizes (2θ = 31.8–38.2°) lie within the angular range covered by the reflections of the standard (30.5–43.6°), so extrapolation was not necessary. The instrumental contribution was removed separately from each component: the instrumental Lorentzian width was subtracted from the measured one, and for the Gaussian component the squares of the widths were subtracted [41,42]. The two corrected components were then combined into the integral breadth of the Voigt profile [42], and this breadth was used in the Scherrer equation. In the single-line method of de Keijser et al., the Lorentzian component is attributed to crystallite size and the Gaussian component to microstrain [41]. Such a separation was not carried out here, and the entire corrected breadth was attributed to crystallite size. The corrected Scherrer sizes are therefore still apparent values and may be underestimated if microstrain is present. Only the corrected sizes are given in the main text. The sizes before and after correction, the data for LaB_6_ and the regression parameters are compiled in the Supplementary Materials (Tables S4–S6) to enable the calculations to be repeated. The values obtained at successive stages of the XRD data processing are compared in Table S7 in the Supplementary Materials.

The FW1/5/4/5M X-ray peak-profile analysis method [43,44,45] was used to estimate an effective mean crystallite size and the width of the associated distribution. Because the reflections sample different crystallographic directions in anisotropic crystallites, the resulting distribution was treated as an effective, orientation-averaged crystallite-size distribution rather than a directly measured particle-size distribution. The analysis was carried out in the Nanopowder XRD Processor Demo program (pre·α ver. 0.0.8, © Pielaszek Research, Warsaw, Poland) with the X10.11a protocol for both series, using the ZnO (100), (002) and (101) reflections jointly. For the ZnO/Ag samples, the analysed data started at 2θ = 28.9–30.2° and ended at 37.6–37.7°, below the Ag (111) reflection at about 38.2°, so the silver phase was not included in the analysis (Table S2 in the Supplementary Materials). The reference series was analysed in the same way, with the data files limited to 2θ = 29.0–38.0° (28.3–37.9° for ZnO-4%H_2_O), so that both series were analysed in comparable windows. The sensitivity of the results to the range of the analysed diffraction pattern was checked on the reference series (Table S2). Changing the range had little effect on the mean sizes, but clearly changed the distribution widths σ. Therefore, the series were compared only in terms of the mean sizes. The FW1/5/4/5M analysis was performed on the measured profiles, without instrumental correction. In its basic form, the method takes into account only the broadening resulting from crystallite size [45]. Because the instrumental broadening was not removed, the FW1/5/4/5M mean sizes may be underestimated, to a greater extent for larger crystallites. The scale of this effect is indicated by the instrumental correction of the Scherrer sizes for the ZnO reflections (Table S6). At nearly matched water content, the reflections of both series have similar widths, so this applies to both series to a similar extent.

### 2.5. Skeletal Density and Specific Surface Area (BET)

Before the measurements, the samples were degassed in a VacPrep 061 station (Micromeritics, Norcross, GA, USA) for 2 h (0.05 mbar, 150 °C). The skeletal (pycnometric) density was determined in accordance with ISO 12154:2014 [46] in an AccuPyc II 1340 helium pycnometer (FoamPyc V1.06, Micromeritics, Norcross, GA, USA) at room temperature. Each density value is the mean of repeated measurement cycles on one specimen, with its standard deviation; these are instrument repetitions and not independent samples. The specific surface area (SSA) was determined in accordance with ISO 9277:2010 [47] on a Gemini 2360 analyser (firmware version 2.01, Micromeritics, Norcross, GA, USA) by nitrogen adsorption (N_2_, 99.999%; Multax, Zielonki-Parcela, Poland), based on the linear form of the Brunauer–Emmett–Teller (BET) isotherm equation in the P/P_0_ range 0.05–0.25. The specific surface area was determined once per sample. The equivalent particle diameter *d*_(BET)_ was calculated from the density and specific surface area, assuming identical, non-porous spherical particles, as *d*_(BET)_ [nm] = 6000/(*ρ*·*S*_BET_) with *ρ* in g/cm^3^ and *S*_BET_ in m^2^/g. Because the density and the specific surface area were measured for the whole nanocomposite, *d*_(BET)_ represents a two-phase model average.

### 2.6. Electron Microscopy (SEM) and Chemical Composition Analysis (EDS)

The morphology of the samples was examined using an ULTRA PLUS scanning electron microscope (SEM) (ZEISS, Oberkochen, Germany), equipped with an immersion-lens detector (InLens), a chamber secondary-electron detector (SE2) and an angle-selective backscattered-electron detector (AsB), which enables compositional contrast to be obtained. Imaging was performed at 2 kV with the InLens detector (working distance ≈3.5 mm), 20 kV with SE2 (≈10 mm) and 8 kV with AsB (≈4.8 mm). AsB images were acquired in compositional-contrast mode. Before the observations, the samples were coated with a thin carbon layer (SCD 005/CEA 035, BAL-TEC, Balzers, Liechtenstein). The chemical composition was analysed by energy-dispersive X-ray spectroscopy (EDS) using a Quantax 400 system (Bruker, Billerica, MA, USA) with an energy resolution of 127 eV and an active area of 30 mm^2^. EDS spectra were recorded at an accelerating voltage of 20 kV, determining zinc and oxygen from the K series and silver from the L series by standardless quantification. Each composition is reported as the mean ± standard deviation of three area analyses at different locations on the powder sample pressed into a pellet.

### 2.7. Comparison of the Series and Data Analysis

The series of ZnO/Ag composites was compared with the Ag-free ZnO reference series in order to check whether the water content affects the ZnO size in the same way in both series. Five quantities were analysed: the specific surface area, *d*_(BET)_, the FW1/5/4/5M mean size and the apparent Scherrer sizes in the a–b plane ((100) reflection) and along the c axis ((002) reflection). The independent variable was the precursor water content measured by the Karl Fischer method (Table 1). Each water content was represented in each series by one synthesis.

(1) Sample pairs. Both series contain samples with similar water content, about 2, 3 and 4 wt%. In these pairs, the relative difference between the composite value and the reference value was expressed as 100 × (ZnO/Ag − ZnO)/ZnO. The samples with the lowest water content (1.12 and 1.47 wt%) were not compared in this way because the difference in water content was too large.
(2) Slopes. A straight line was fitted to the four points of each series by the least-squares method. The slope b corresponds to the mean change in the given quantity for an increase in water content of 1 wt%. The difference between the slopes of the two series was expressed as 100 × |b(ZnO/Ag) − b(ZnO)|/|b(ZnO)|. For the c axis, where one pair of samples deviated from the others, the slopes were additionally determined without this pair.
(3) Deviations from the reference series. The water contents in the two series are not identical. The reference value at the water content of a given composite was determined by linear interpolation between the two adjacent points of the reference series. For 3.97 wt%, a short linear extrapolation from the last two points was used. The relative deviations of the four composite samples were summarised by the root mean square (RMS).
(4) Increase over the common range. The relative increase in *d*_(BET)_ was calculated for both series over the water content range of 1.47–3.96 wt%, common to both series. The reference value at 1.47 wt% was determined by interpolation, as in point (3). This increase was compared with the differences between the series from point (1).

The distribution widths σ from the FW1/5/4/5M method were omitted from the comparison because they depend on the range of the analysed diffraction pattern (Table S2). The scatter between syntheses could not be estimated, and no statistical tests were applied. The reported standard deviations reflect the variability of repeated determinations on the same precursor or sample, rather than variability between independent syntheses. The differences given refer to individual samples and are not measurement uncertainties. The calculations were performed in an Excel spreadsheet (version 2608, build 16.0.20326.20072, 64-bit, Microsoft Corporation, Redmond, WA, USA). The formulae and all values are compiled in Table S3.

## 3. Results and Discussion

### 3.1. Phase Composition and Crystallite Size

**Figure 1:**
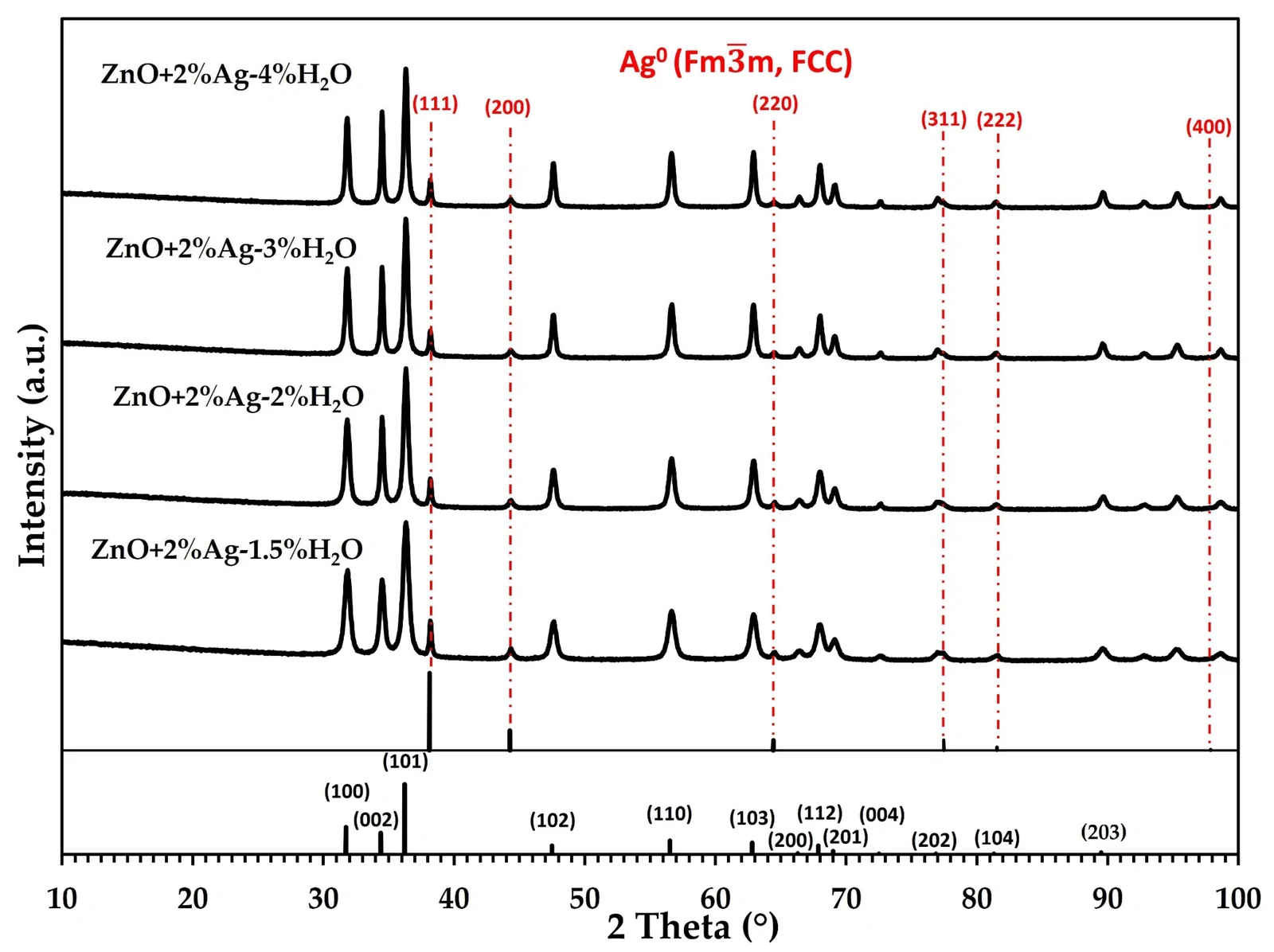
X-ray diffraction (XRD) patterns of ZnO/Ag nanocomposites obtained by MSS at nominal precursor water contents of 1.5, 2, 3 and 4 wt% (nominally 2 mol% Ag). Numbers in parentheses denote Miller indices. Red dashed lines mark Ag reflections. The bottom bars show Ag and ZnO reference reflections.

The diffraction patterns of all four samples (Figure 1) show the reflections of two crystalline phases: hexagonal ZnO wurtzite (space group P6_3_mc) and face-centred cubic (FCC) metallic silver Ag^0^ (Fm $\bar{3}$ m). The positions and relative intensities of the ZnO reflections, including (100), (002), (101), (102), (110), (103), (112) and (201), correspond to the reference wurtzite structure. No additional crystalline phases beyond ZnO and Ag, including Ag_2_O or AgO [48], were detected; a thin surface oxide or X-ray-amorphous Ag species cannot be excluded [49]. Two-phase ZnO/Ag composites in which only the wurtzite and cubic-silver reflections are recorded have likewise been reported [20,21,50].

In the 2θ ≈ 35–47° range the silver (111) (≈38.2°) and (200) (≈44.4°) reflections are clearly separated from the adjacent ZnO (101) and (102) reflections, which confirms a separate crystalline metallic Ag phase at a nominal silver content of ≈2 mol%. XRD neither quantifies the fraction of the total Ag in this phase nor excludes minor lattice-incorporated or X-ray-amorphous Ag. In the sense of the distinction drawn in the Introduction [3,4], the materials are two-phase ZnO/Ag^0^ nanocomposites rather than conventionally Ag-doped ZnO.

The formation of XRD-detectable metallic Ag^0^ without a separately added reducing agent is consistent with reports of Ag^+^ reduction by ethylene glycol under heated polyol conditions [28,51,52,53].

The Ag^+^ ion is one of the readily reducible metal cations, as indicated by its standard potential in aqueous solution (Ag^+^/Ag^0^ ≈ +0.80 V versus the standard hydrogen electrode) [28]. In the polyol method, the silver precursor first dissolves, after which the metallic phase nucleates and grows directly in the solution, without a solid intermediate phase [28,52]. The pathway of Ag^0^ formation therefore differs from the pathway of ZnO formation, which in glycol proceeds via basic zinc double salts and esterification [25,26]. Ethylene glycol is oxidised in two-electron steps to glycolaldehyde and further to glycolic acid, and in each step releases two protons [28]. Combining these steps with the half-reaction Ag^+^ + e^−^ → Ag^0^ gives Equations (2) and (3):

2 Ag^+^ + HOCH_2_–CH_2_OH → 2 Ag^0^ + HOCH_2_–CHO + 2 H^+^ (2)

2 Ag^+^ + HOCH_2_–CHO + H_2_O → 2 Ag^0^ + HOCH_2_–COOH + 2 H^+^ (3)

For silver, it has been shown that the main reducing agent is glycolaldehyde formed from glycol [28], which corresponds to Equation (3). Glycolaldehyde may also be oxidised by another route, to glyoxal [28]. The Ag particles being formed may additionally catalyse the oxidation of glycol [28]. Ag particles may form in ethylene glycol even at room temperature [53], and syntheses of silver in polyols are usually carried out at a temperature of at least 100 °C [28]. In this work, the MSS temperature was 220 °C. As long as the metal particles remain in the polyol, this environment protects them from oxidation [28]. No crystalline silver oxides were detected in the diffraction patterns. Equations (2) and (3) describe the reduction pathway adopted on the basis of the literature, without a direct investigation of the mechanism in this work.

**Figure 2:**
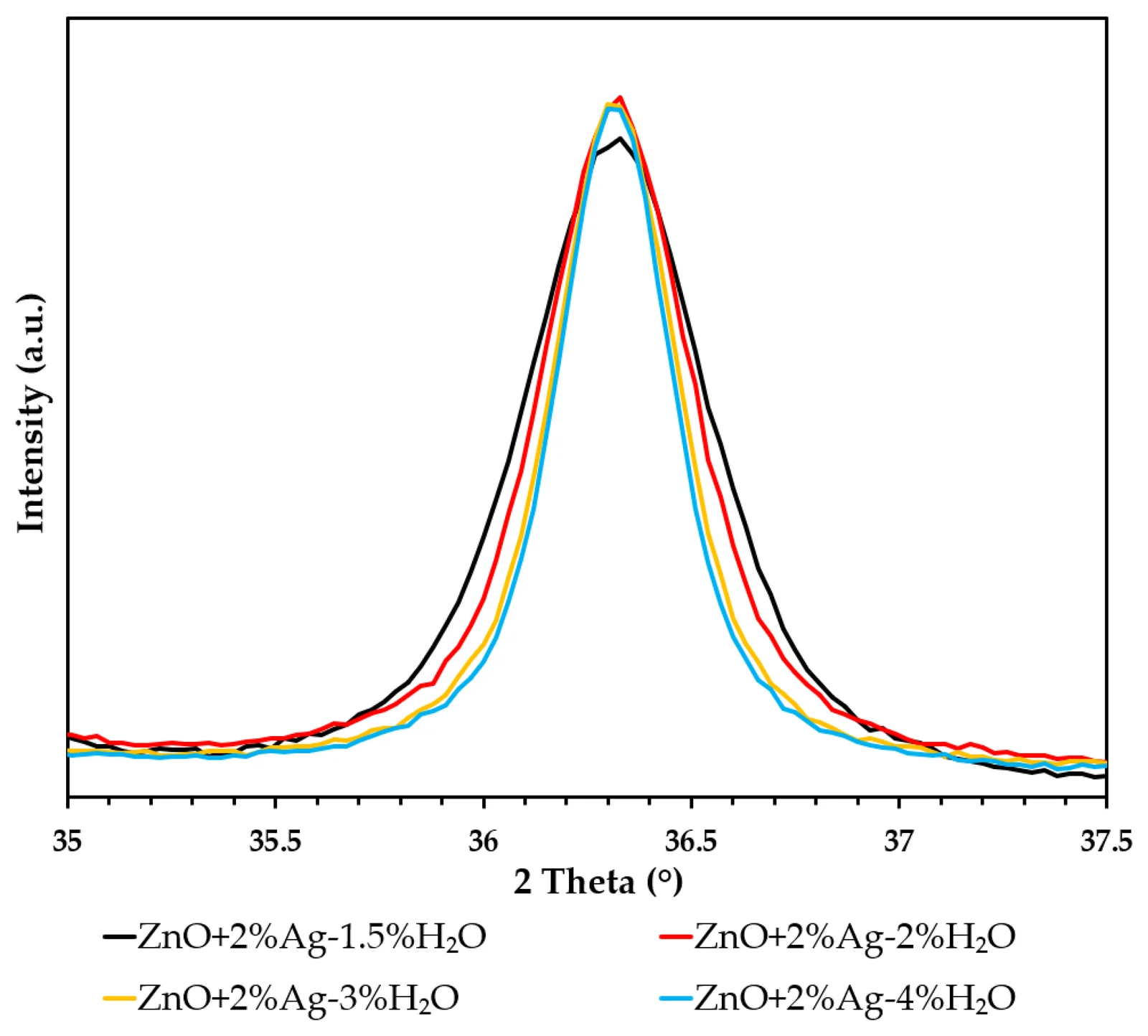
X-ray diffraction (XRD) patterns of ZnO/Ag nanocomposites obtained by MSS, shown enlarged over the 2θ angle range from 35° to 37.5° covering the (101) reflection of ZnO wurtzite, at nominal precursor water contents of 1.5, 2, 3 and 4 wt% (nominally 2 mol% Ag). The profiles are plotted in arbitrary units on a common intensity scale.

The apparent ZnO crystallite size increased monotonically with the measured precursor water content. In the basal plane, it increased from 15 nm at 1.47 wt% H_2_O to 25 nm at 3.97 wt%, and along the c axis from 18 to 40 nm (Table 2). The progressive narrowing of the ZnO reflections (Figure 2) qualitatively supports this trend. The direction of this trend agrees with earlier pure-ZnO and two-point ZnO/Ag studies [25,26,38], and the present series extends the observation across four water contents. The matched Ag-free ZnO reference series is compared with the composites in Section 3.3. A decrease in ZnO crystallite size (from 27 to 20 nm at 0–10 wt% Ag) and broadening of the ZnO reflections with increasing silver content have been reported for Ag-modified ZnO prepared by aqueous precipitation [54]. The nominal Ag content was constant in all composite samples and amounted to 2 mol%. In Section 3.3, this series is compared with Ag-free ZnO. However, no series with different Ag contents, for example 1, 2 and 4 mol%, was prepared. The data cover only two Ag contents, 0 and 2 mol%, originating from separately prepared series. They are insufficient to determine whether the ZnO crystallite size changes with the Ag content, as described for ZnO obtained by the precipitation method [54].

The data presented show that the apparent ZnO crystallite size and *d*_(BET)_ increase with the measured precursor water content. The mechanism of this effect was not investigated in this work. The dependence of the ZnO particle size on the precursor water content was described for this method in [25], and its mechanism was explained and verified experimentally in [26]. Water causes hydrolysis of zinc acetate, in which acetic acid is formed. The intermediate product Zn_5_(OH)_8_(CH_3_COO)_2_·xH_2_O is formed in the reactions of zinc acetate with ethylene glycol, simultaneously with the esterification of acetic acid with glycol, in which esters and water are formed. Because water is a product of esterification, its higher initial content means that the esterification equilibrium is reached with a smaller amount of the intermediate product. Once equilibrium has been reached, the intermediate product decomposes into ZnO nuclei with a size of about 16 nm. New nuclei no longer form, and the existing ones grow until the unreacted zinc acetate is exhausted. At a higher water content, therefore, fewer nuclei form and more zinc acetate remains for their growth, which is why the final particles are larger. After the reactants were exhausted, the particles did not grow further and no Ostwald ripening was found [26]. The results obtained in this work for ZnO/Ag are consistent with this mechanism, but do not constitute an independent test of it in the presence of Ag. All precursors were prepared with the same amount of zinc per portion. The ZnO particle size also did not depend on the zinc acetate concentration at constant water content [26]. The yield of this synthesis for pure ZnO was 94–98% at 1.5 and 4 wt% H_2_O [26]. For ZnO obtained from zinc acetate in primary alcohols, yields of 95–97% were reported [35]. If the yield of the composites was similarly high, larger crystallites imply fewer crystallites, which is consistent with this mechanism. Neither the number of nuclei nor the course of growth was measured in this work, because the products were examined only after 25 min of synthesis. A smaller number of larger crystallites would, however, also result from their coarsening after nucleation. For ZnO obtained from zinc acetate in 2-propanol, the increase in particle size with water content was attributed precisely to faster diffusion-controlled coarsening [34]. Such coarsening was not investigated in the present work. The direction of the dependence of size on water content therefore does not by itself establish the growth mechanism. The solvent itself also affects growth: it has been proposed that in primary alcohols adsorbed alkyl chains restrict the growth of ZnO in specific directions and in this way change the shape of the particles [35].

In all samples the ZnO crystallites are anisotropic, larger along the c axis than in the a–b basal plane (c/(a–b) ratio ≈ 1.2–1.6; Table S8), which reflects the preferential growth of wurtzite along the polar c axis; the degree of this anisotropy depends on the solution chemistry, e.g., on the dissolved Zn species [55,56]. This anisotropy is maintained across the whole range of water content studied. The instrumental correction slightly increased the c/(a–b) ratios compared with the uncorrected values (1.2–1.5; Tables S6 and S8).

**Figure 3:**
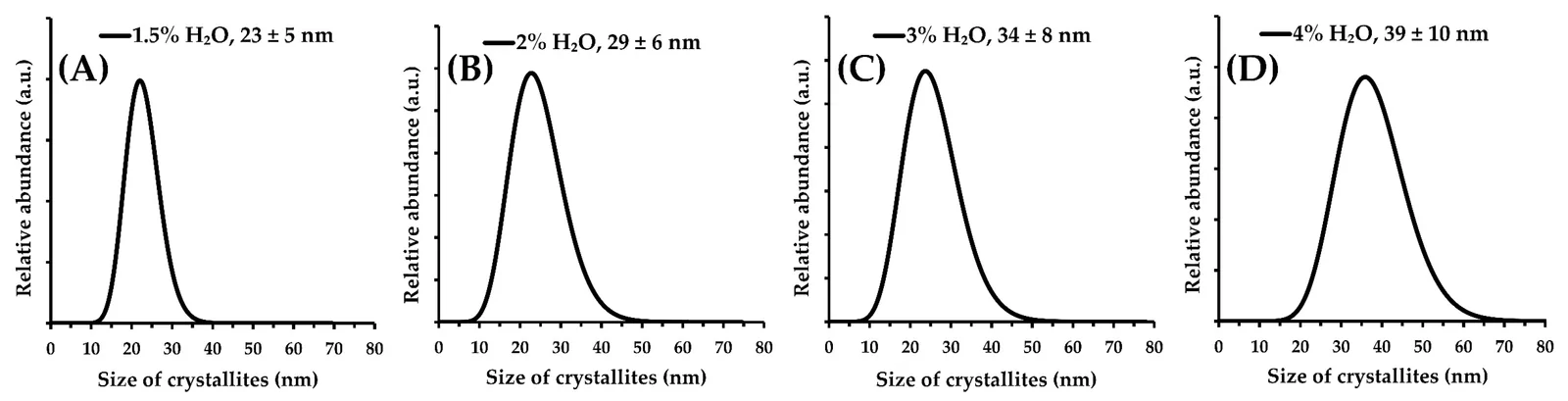
Effective crystallite-size distributions of ZnO/Ag nanocomposites determined from the diffraction line profile by the FW1/5/4/5M method (Nanopowder XRD Processor Demo, pre α ver.0.0.8, © Pielaszek Research) [43,44,45]: (**A**) 1.5% H_2_O, (**B**) 2% H_2_O, (**C**) 3% H_2_O, (**D**) 4% H_2_O.

The FW1/5/4/5M analysis yielded an effective mean crystallite size and an associated distribution width σ (Figure 3). The mean size increased from 23 to 39 nm, in agreement in direction with the sizes determined by the Scherrer method, while the distribution width σ increased from 5 to 10 nm, so that the relative width stayed approximately constant (σ/d ≈ 0.21–0.27). The modelled distributions therefore shifted towards larger sizes without an appreciable change in their relative width. A comparable relative width of the FW1/5/4/5M distribution (σ/d ≈ 0.24–0.30) and a narrow TEM particle-size distribution were reported for MSS ZnO obtained at 1–4 wt% water [57], although particle-size and effective crystallite-size distributions are not directly equivalent.

The apparent Ag^0^ crystallite sizes, determined from the (111) reflection, were 41, 41, 39 and 40 nm at a nominal water content of 1.5, 2, 3 and 4 wt%. Because the Ag (111) reflection was weak at the low nominal silver content used (2 mol%) and no uncertainty was quantified for these apparent sizes, the small differences between these values are not interpreted as evidence of a dependence on water content. The instrumental correction was also largest for this reflection (18–20%), so the Ag^0^ sizes depend on the correction more than the ZnO sizes do (Table S6). The apparent values are consistent with Ag crystallites of the same order as, or larger than, those of ZnO.

### 3.2. Specific Surface Area and Skeletal Density

**Figure 4:**
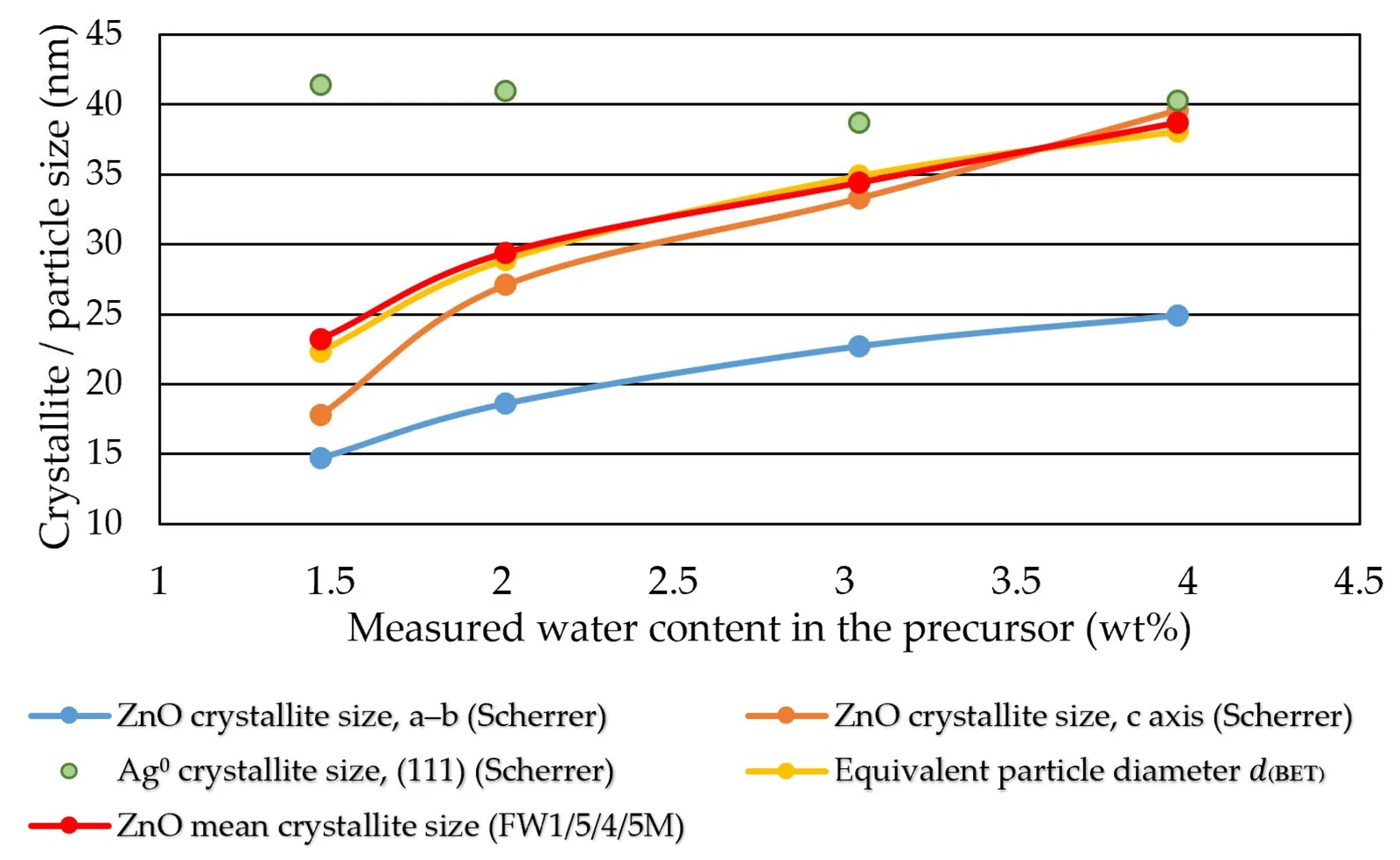
Apparent ZnO crystallite sizes calculated from the Scherrer equation on the basis of the (100) reflection (a–b basal plane) and the (002) reflection (c axis), the effective mean FW1/5/4/5M crystallite size, the equivalent particle diameter *d*_(BET)_, and the apparent Ag^0^ crystallite size from the (111) reflection (points only), plotted against the measured (Karl Fischer) precursor water content.

The specific surface area decreased from 50.7 to 29.0 m^2^/g, while the equivalent diameter *d*_(BET)_ increased from 22 to 38 nm (Table 3). The *d*_(BET)_ values were close to the FW1/5/4/5M mean size (23–39 nm; Figure 3, Table 2) and in all samples exceeded the Scherrer sizes in the a–b basal plane (15–25 nm). Along the c axis (18–40 nm), *d*_(BET)_ was larger in the three samples with the lowest water content, and at 3.97 wt% H_2_O the two values were similar (38.1 and 39.6 nm). These results are not fully independent. The *d*_(BET)_ value was calculated from the BET specific surface area and the measured density, so it is not independent of the specific surface area. The Scherrer and FW1/5/4/5M sizes, in turn, come from the same diffraction patterns. The comparison therefore covers two independent methods, XRD and BET, and not four independent confirmations of the trend. The close numerical agreement among the methods is consistent with, but does not prove, particles dominated by a single crystallite, with limited permanent aggregation; a similar interpretation was supported by the agreement of TEM, BET and XRD sizes for ZnO obtained by MSS at 1–4 wt% water [57]. A similar agreement between TEM particle sizes and XRD crystallite sizes was described for ZnO obtained from zinc acetate in alcohols [35,36]. For ZnO obtained by forced solvolysis in ethylene glycol, the mean particle size from TEM (44 nm) corresponded to the crystallite size from Rietveld refinement (45 nm), and the particles were described as single crystals [36]. Direct confirmation for the present samples would require TEM or HRTEM [25,26,27]. The sizes determined from XRD and the equivalent diameter *d*_(BET)_ describe different features of the material. The Scherrer equation with the integral breadth at K = 1 gives the volume-weighted mean crystallite thickness, measured perpendicular to the reflecting planes, and not its diameter (largest thickness) [40]. Unlike the size calculated from the FWHM, it has a clear physical interpretation [40]. The *d*_(BET)_ value, by contrast, is calculated from the density and the surface area accessible to the adsorbate, assuming identical, non-porous spherical particles. If a crystallite were a sphere, its diameter would be 4/3 of this mean thickness [40]. After conversion in this way, the Scherrer sizes are 20–33 nm along a–b and 24–53 nm along c. The equivalent diameter *d*_(BET)_ (22–38 nm) lies between these two values in each sample. The conversion, however, assumes a spherical shape, as does the determination of the equivalent diameter *d*_(BET)_, whereas the ZnO crystallites are elongated along the c axis. The similar values of *d*_(BET)_ and of the crystallite sizes suggest that most particles are single crystallites. These data do not exclude the presence of particles composed of two or several crystallites. Figure 4 compares all four size measures against the measured precursor water content.

The skeletal densities (5.32–5.43 g/cm^3^) were below that of bulk wurtzite ZnO (5.61 g/cm^3^). They were also below the rule-of-mixtures value calculated for the nominal ZnO/Ag composition (~5.68 g/cm^3^, from 5.61 g/cm^3^ for ZnO and 10.49 g/cm^3^ for metallic Ag at about 2.6 wt% Ag). The skeletal density increased slightly across the series. Surface-associated species may contribute to this difference. For MSS ZnO, a density increase with particle size was attributed to a thinner disordered surface shell observed by TEM [57], and a similar size dependence was reported for ZrO_2_ nanopowders [58]. Defects and closed porosity are additional possibilities but were not examined here.

### 3.3. Comparison with the Ag-Free ZnO Reference Series

**Figure 5:**
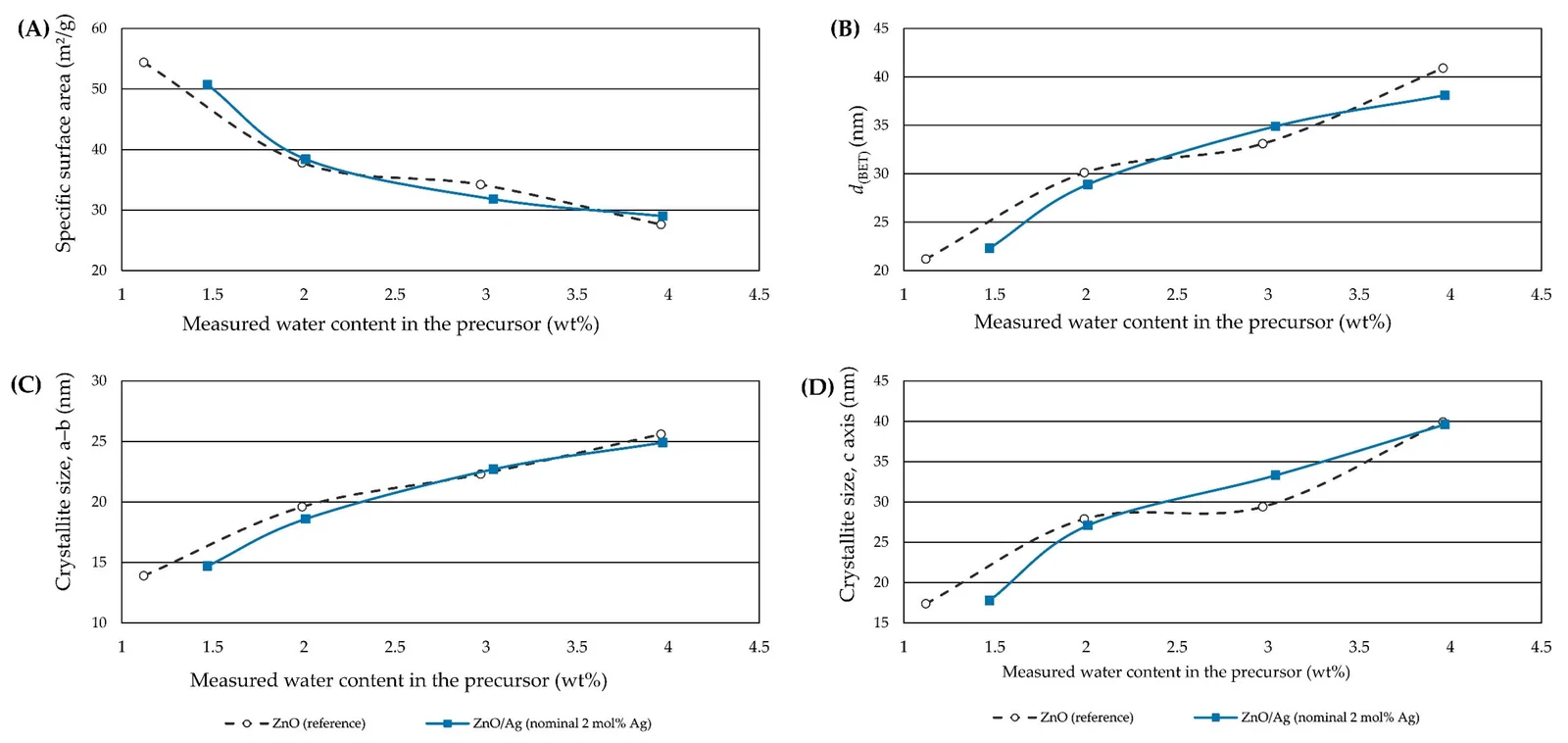
Comparison of the Ag-free ZnO reference series and the ZnO/Ag series (nominally 2 mol% Ag) as a function of the measured precursor water content: (**A**) BET specific surface area; (**B**) equivalent diameter *d*_(BET)_; (**C**) apparent ZnO crystallite size in the a–b basal plane, from the (100) reflection; (**D**) apparent ZnO crystallite size along the c axis, from the (002) reflection.

The Ag-free ZnO reference series (Section 2.2) covers a range of measured precursor water content that partly overlaps with the range of the ZnO/Ag series, and was characterised by the same methods (Table 4; Figures S1–S3 and Table S1 in the Supplementary Materials). In the range from 1.12 to 3.96 wt% H_2_O, the specific surface area decreased from 54.4 to 27.6 m^2^/g, the equivalent diameter *d*_(BET)_ increased from 21 to 41 nm, and the FW1/5/4/5M mean size increased from 21.5 to 40.1 nm. The directional Scherrer sizes increased from 14 to 26 nm in the a–b basal plane and from 17 to 40 nm along the c axis. The direction and scale of these changes are similar to those recorded for the composites (Table 2 and Table 3). In Figure 5, both series are shown on common axes.

At nearly matched measured water content, both series give similar values (Figure 5A,B). At 1.99 and 2.01 wt% H_2_O, the specific surface areas were 37.8 and 38.4 m^2^/g, the equivalent diameters *d*_(BET)_ 30.1 and 28.9 nm, and the FW1/5/4/5M mean sizes 31.7 and 29.4 nm. At 3.96 and 3.97 wt%, the corresponding values were 27.6 and 29.0 m^2^/g, 40.9 and 38.1 nm, and 40.1 and 38.7 nm. Including the pair at 2.97 and 3.04 wt% H_2_O, the largest relative difference for any of these quantities is 7.3%. The specific surface area and *d*_(BET)_ do not depend on the XRD data. The FW1/5/4/5M mean size is treated here only as an auxiliary measure, for the reasons given in Section 2.4. The lowest water contents, 1.12 and 1.47 wt%, do not form a matched pair. In Figure 5, the first points of the two series are therefore offset relative to each other along the water content axis, and their vertical distance should not be read as a difference between the series.

The slopes of the straight lines fitted to the results of both series give the change in a given quantity for an increase in water content of 1 wt%. For the reference series and the composite series, respectively, they are:

- specific surface area: −8.75 m^2^/g and −8.01 m^2^/g;
- *d*_(BET)_: +6.51 nm and +6.09 nm;
- FW1/5/4/5M mean size: +6.09 nm and +5.89 nm.

The absolute values of the slopes for the composite series are 8.4%, 6.4% and 3.2% lower than those for the reference series. The root mean square (RMS) deviations of the composite values from the reference series are 5.1%, 6.7% and 6.3%. The largest deviation, 10.0% for *d*_(BET)_, occurred at the lowest water content. In this range, *d*_(BET)_ of the reference series changes most strongly, from 21.2 to 30.1 nm. All values are compiled in Table S3.

The Scherrer sizes in both directions increased with water content in both series. In the a–b basal plane, the slopes are +3.96 and +3.98 nm per wt% (difference 0.6%), and the differences within the sample pairs lie in the range 1.8–5.1%. Along the c axis, the slopes are +7.24 and +8.16 nm per wt% (difference 12.8%). The differences within the pairs at about 2 and 4 wt% H_2_O are 2.9 and 0.8%, and 13.3% at about 3 wt%. After omitting this pair, the difference between the slopes is 6.3%. In the reference series, between 1.99 and 2.97 wt% H_2_O, the size along the c axis increased by 5% (from 27.9 to 29.4 nm), and in the a–b plane by 14% (from 19.6 to 22.3 nm). In the composites, over the same interval, the size along the c axis increased by 23% (from 27.1 to 33.3 nm). The difference in the pair at about 3 wt% therefore results mainly from the size along the c axis for the ZnO-3%H_2_O sample. On the basis of the present data, it cannot be determined whether the cause lies in the properties of this sample or in the fitting of the (002) reflection. For this reason, the comparison along the c axis is less certain than in the a–b plane. The instrumental correction is largest for the narrow (002) reflections. Before correction, the slopes along the c axis differed by 10.8% (Table S3). The c/(a–b) ratio increased in both series from about 1.2–1.3 to about 1.6 (Table S8). Since it changed similarly in both series, the data do not indicate that Ag affected the anisotropy of the ZnO crystallites.

Over the full water content ranges, *d*_(BET)_ increased by 71% in the composites and by 93% in the reference series. Over the common range of 1.47–3.96 wt%, the increases are about 71% and 65%. They are about ten times larger than the largest difference in *d*_(BET)_ within the sample pairs (6.8%). The differences between the series are small compared with the changes associated with water content (Figure 5). The similar slopes describe the measured points, but do not demonstrate the equivalence of the two systems.

The only difference in the composition of the precursors of the two series was the addition of silver(I) acetate. The zinc concentration in both series was 0.304 mol/L. Silver acetate increased the total amount of acetate ions by about 1%, and the amount of metal cations by about 2%. In earlier work [26], a threefold change in zinc acetate concentration (0.12–0.36 mol dm^−3^) at a constant water content of 1.5 wt% did not significantly change the particle size of pure ZnO. The *d*_(BET)_ values were in the range 27–30 nm, and the specific surface area in the range 38.5–42.3 m^2^/g [26]. The change introduced by silver acetate is much smaller, and the zinc concentration used in this work (0.304 mol/L) is equal to one of the levels studied in [26]. The change in the amount of acetate alone should therefore not significantly affect the ZnO size. This argument does not cover the effect of Ag^+^ and Ag^0^. The independence of particle size from precursor concentration is not a general feature of the polyol method. For Ni and Co, the particle size increased with increasing amount of reactant relative to the polyol [52]. For ZnO, this independence follows from the mechanism described in [26]. Ethylene glycol is present in a large excess relative to zinc acetate, and therefore, at constant water content, a constant fraction of zinc acetate reacts until the esterification equilibrium is reached. The number of ZnO nuclei then increases in proportion to the amount of acetate, as does the amount of acetate remaining for their growth. The amount of ZnO obtained increases, while the particle size remains the same [26]. The argument concerning silver acetate is based on this mechanism and on the data for ZnO [26].

Equations (2) and (3) show that one proton is released for each Ag^+^ ion reduced. With acetate ions, these protons form acetic acid. Acetate ions may also partially deprotonate glycol [28]. Esterification of acetic acid with glycol leads to the formation of water [26]. With complete reduction of Ag^+^ and complete esterification, the amount of this water would not exceed about 0.01 wt%. This is less than the standard deviation of the Karl Fischer determinations (Table 1).

Readily reducible silver forms particles in polyols earlier than other metals and may act as a nucleus for their growth [52]. The addition of silver nitrate at an Ag/metal molar ratio of 10^−4^–10^−2^ gave smaller and uniform Co and Ni particles, whose diameter decreased with increasing amount of Ag [52]. In ZnO modified with silver by the precipitation method, the reduction in size was attributed to Ag clusters acting as nucleation centres [54]. In this work, the Ag/Zn ratio was about 2 × 10^−2^. The similar final ZnO sizes in both series do not, however, show whether the Ag particles took part in nucleation. The scale of a possible effect can only be estimated in a simplified model. The estimate assumed spherical, identical particles of each phase, complete conversion of the precursors, and the *d*_(BET)_ of the composite as an approximation of the ZnO particle diameter, which is justified by the small proportion of Ag (about 2.6 wt%). The Ag particles were assumed to have the Ag crystallite size determined from XRD (about 40 nm, Table 2). This is an approximation, because the Ag particles may be polycrystalline and their sizes may have a broad distribution. With complete conversion of the precursors, there are about 80–400 ZnO particles with a size of 22–38 nm per Ag particle. If each final Ag particle corresponded to one additional ZnO nucleation centre (a similar assumption was made for Co and Ni [52]), the mean ZnO size would decrease by less than 0.5%. The model does not, however, determine the actual contribution of Ag. The number and size of the nucleation centres during synthesis were not measured, and one Ag particle may have initiated more than one ZnO particle. Moreover, ZnO is formed as a result of hydrolysis and esterification, unlike the metals described in [52].

The limitations of the comparison result from the way in which the reference series was prepared (Section 2.2). The precursors of the two series were prepared separately, and the syntheses were performed ten days apart. Each water content is represented by one synthesis, so the scatter between syntheses remains unknown. The differences between the series may be affected by the presence of Ag, impurities introduced with silver(I) acetate, the separate preparation of the precursors and the scatter between syntheses. The present experimental design does not allow these contributions, which may add up or partly compensate one another, to be separated.

### 3.4. Elemental Composition and Morphology

**Figure 6:**
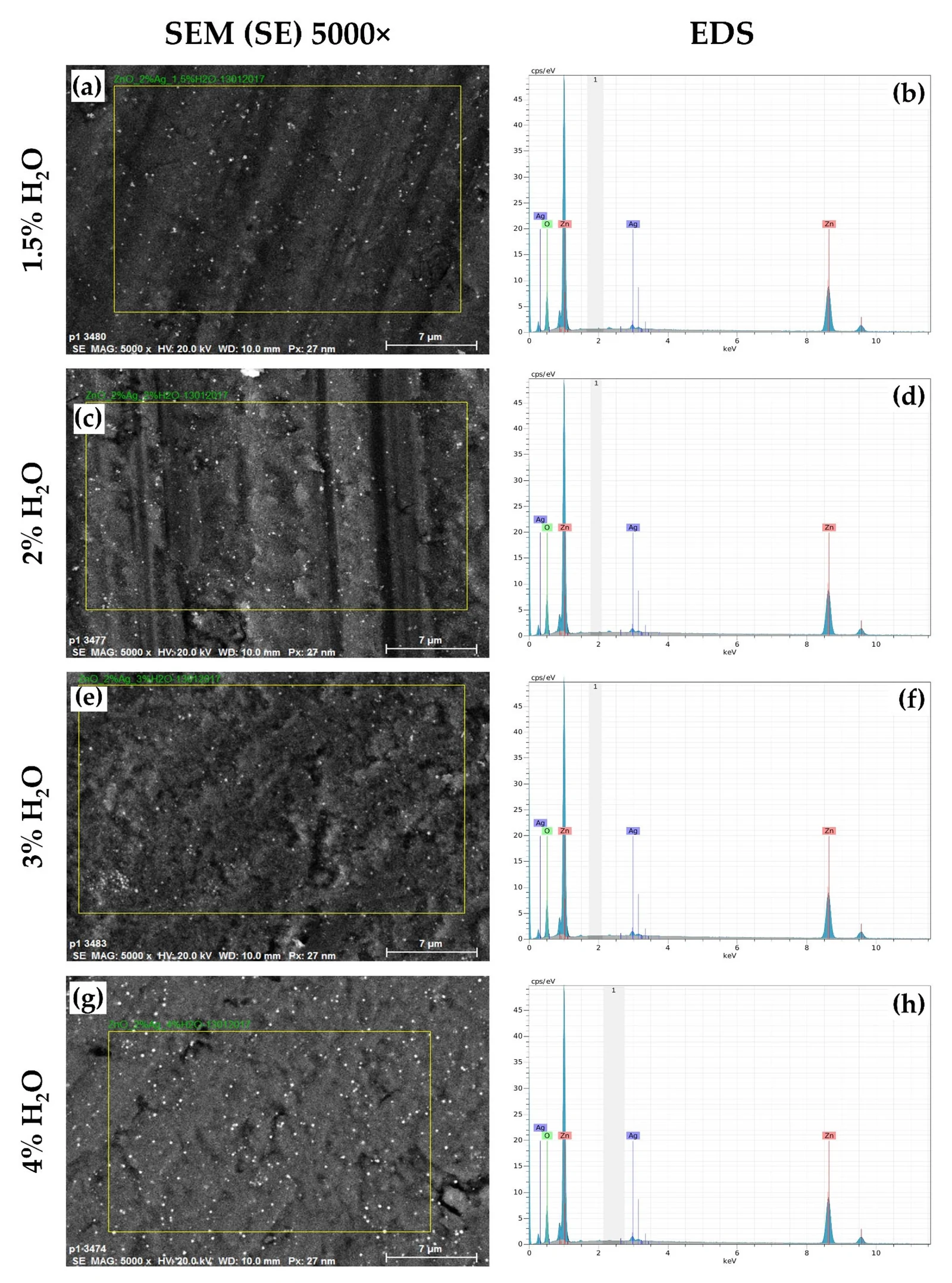
SEM images (chamber secondary-electron detector, SE2; 5000×) and the corresponding EDS spectra of ZnO/Ag nanocomposites at nominal precursor water contents of 1.5, 2, 3 and 4 wt%: (**a**,**c**,**e**,**g**) microstructure, with the EDS analysis area marked by a yellow frame; (**b**,**d**,**f**,**h**) the EDS spectra from these areas.

Representative SEM images (chamber secondary-electron detector, SE2) together with the corresponding EDS spectra for all four samples are presented in Figure 6. The images show bright objects, tens to more than 100 nm in size, dispersed within the compact matrix of nanometric ZnO. Because SE contrast is mainly topographic, these objects cannot be assigned to silver from the SE images alone. Each spectrum recorded from the marked areas contained Zn, O and Ag lines, including a distinct Ag Lα peak at ≈3 keV. Apart from carbon from the conductive carbon coating, which was excluded from composition normalisation, no other elements were detected within the sensitivity of EDS.

The average elemental composition determined by EDS is summarised in Table 5. In all samples the matrix is dominated by zinc and oxygen, consistent with the presence of the ZnO phase; because of the semi-quantitative nature of EDS for oxygen, the O/Zn values should be treated as approximate. The area-averaged estimates of the Ag content were similar for the four samples (2.12–2.20 wt%; 0.82–0.86 at%) and showed no monotonic trend with water content. Because the measurements were standardless and limited to three fields per sample, they describe the composition of the analysed regions and do not establish the bulk Ag content or exclude differences between samples.

Conversion of the EDS results to a Zn + Ag basis gave approximately 1.6 mol% Ag, compared with the nominal 2.0 mol%. This difference is not sufficient evidence of Ag loss and does not exclude a real deviation of the composition from the nominal value: the estimate rests on three standardless area analyses and is affected by systematic quantification error [59] and by spatial sampling of a sparse Ag phase. A bulk Ag balance would require ICP-OES or ICP-MS after sample dissolution. All four precursors were taken from one solution and had the same nominal Ag/(Ag + Zn) ratio. The Ag content in the products may, however, have differed if the reduction and retention of Ag depended on the water content. The area-averaged EDS estimates show no monotonic trend (Table 5). EDS analysis is semi-quantitative and does not exclude differences in the bulk Ag content. The dependence of size on water content was discussed without assuming that the actual Ag content was the same in all samples. The Ag-free reference series shows a similar dependence on water content (Section 3.3). The increase in crystallite size with water content therefore also occurs without the involvement of silver. The data do not, however, rule out the possibility that the Ag content or distribution affected the sizes obtained in the individual composites.

**Figure 7:**
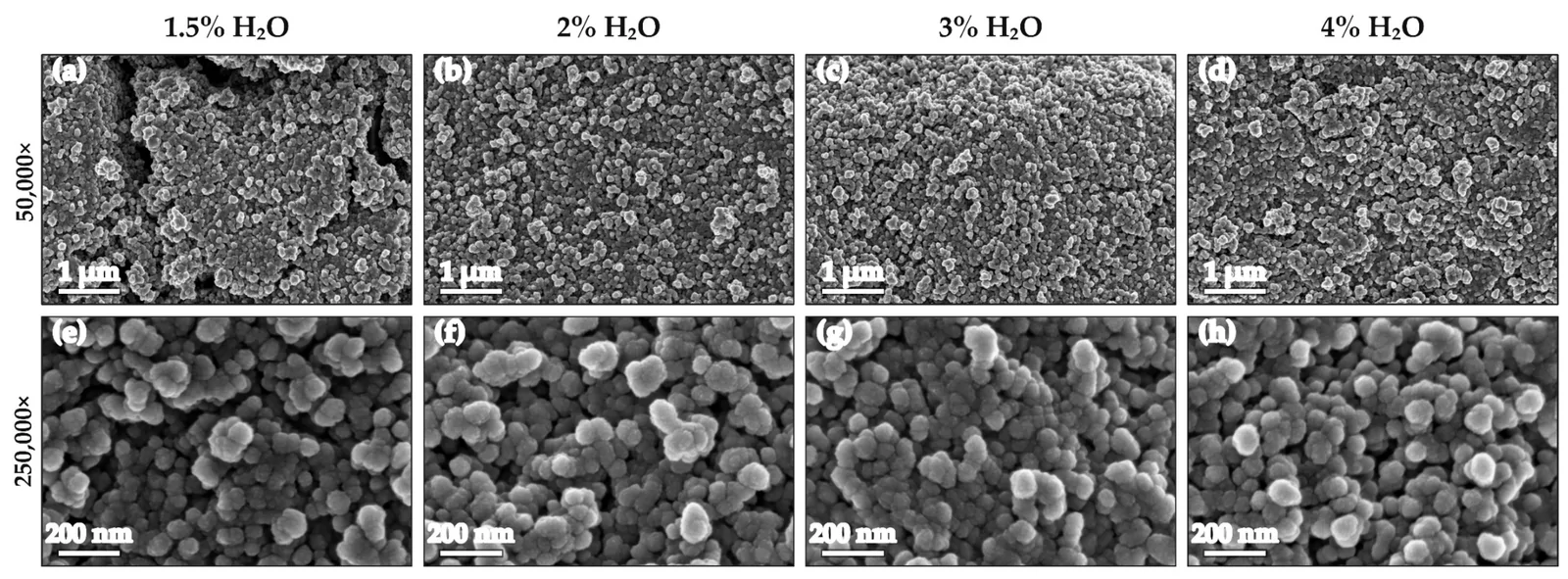
SEM images (InLens detector, secondary electrons) of ZnO/Ag^0^ nanocomposites at nominal precursor water contents of 1.5, 2, 3 and 4 wt% (nominally 2 mol% Ag): (**a**–**d**) 50,000×, microstructure overview; (**e**–**h**) 250,000×, nanoparticles. Columns correspond to successive water contents ((**a**,**e**): 1.5; (**b**,**f**): 2; (**c**,**g**): 3; (**d**,**h**): 4 wt%).

High-resolution SEM imaging (Figure 7) complements the microstructure picture from Figure 6. At a magnification of 50,000× (Figure 7a–d), all four samples form a compact layer composed of morphologically similar regions, without extensive zones of a different ZnO nanoparticle structure. At 250,000× (Figure 7e–h) the samples contain rounded, slightly elongated particles with apparent dimensions of about 20–50 nm, which is qualitatively consistent with the moderate crystallite anisotropy determined by XRD (c/(a–b) ≈ 1.2–1.6). The particles remain distinguishable within loosely packed, irregular clusters. This is a morphology typical of nanopowders obtained by the MSS method [25,26,27]. The SEM particle dimensions are of the same order as *d*_(BET)_ and the XRD-derived sizes (Table 2). This supports, but does not confirm, the interpretation that many particles contain a single dominant crystallite [25,26,27].

According to ISO terminology, the loose clusters of distinguishable particles seen here are more consistent with agglomerates than with permanently fused aggregates [27,60]; SEM alone, however, does not establish the strength of the interparticle bonding. The similarity between *d*_(BET)_ and the XRD-derived sizes (Table 2) is also consistent with limited permanent aggregation, since permanent fusion would reduce the surface accessible to the adsorbate. In related MSS ZnO powders, aggregate size depended on the microwave power during synthesis, and uncontrolled agglomeration and aggregation were reported to occur most often during washing and drying [27]. Freeze-drying was therefore used here to limit changes during drying.

No clear particle-size trend could be resolved visually in the SEM images. No quantitative analysis of particle size was performed on the SEM images. The particle boundaries in the loose clusters were not sufficiently distinct, and the differences between the samples were masked by the surface texture. The quantitative comparison therefore relies on the XRD and BET results (Table 2 and Table 3). The morphology is similar across the whole series, and the area-averaged EDS estimates of the analysed fields are comparable (Table 5). The differences in apparent powder-layer thickness likely reflect specimen preparation rather than intrinsic differences between the materials.

Silver was assessed by area-averaged EDS for all four samples and by AsB Z-contrast imaging for one sample. Three area analyses per sample gave comparable mean Ag estimates of about 2.1–2.2 wt% (Table 5). In the SEM images, bright objects are dispersed in the ZnO matrix within the fields of view analysed, and a few small clusters of brighter particles occur locally (Figure 7). SE images provide morphological rather than compositional contrast and therefore cannot be used to assess Ag segregation.

**Figure 8:**
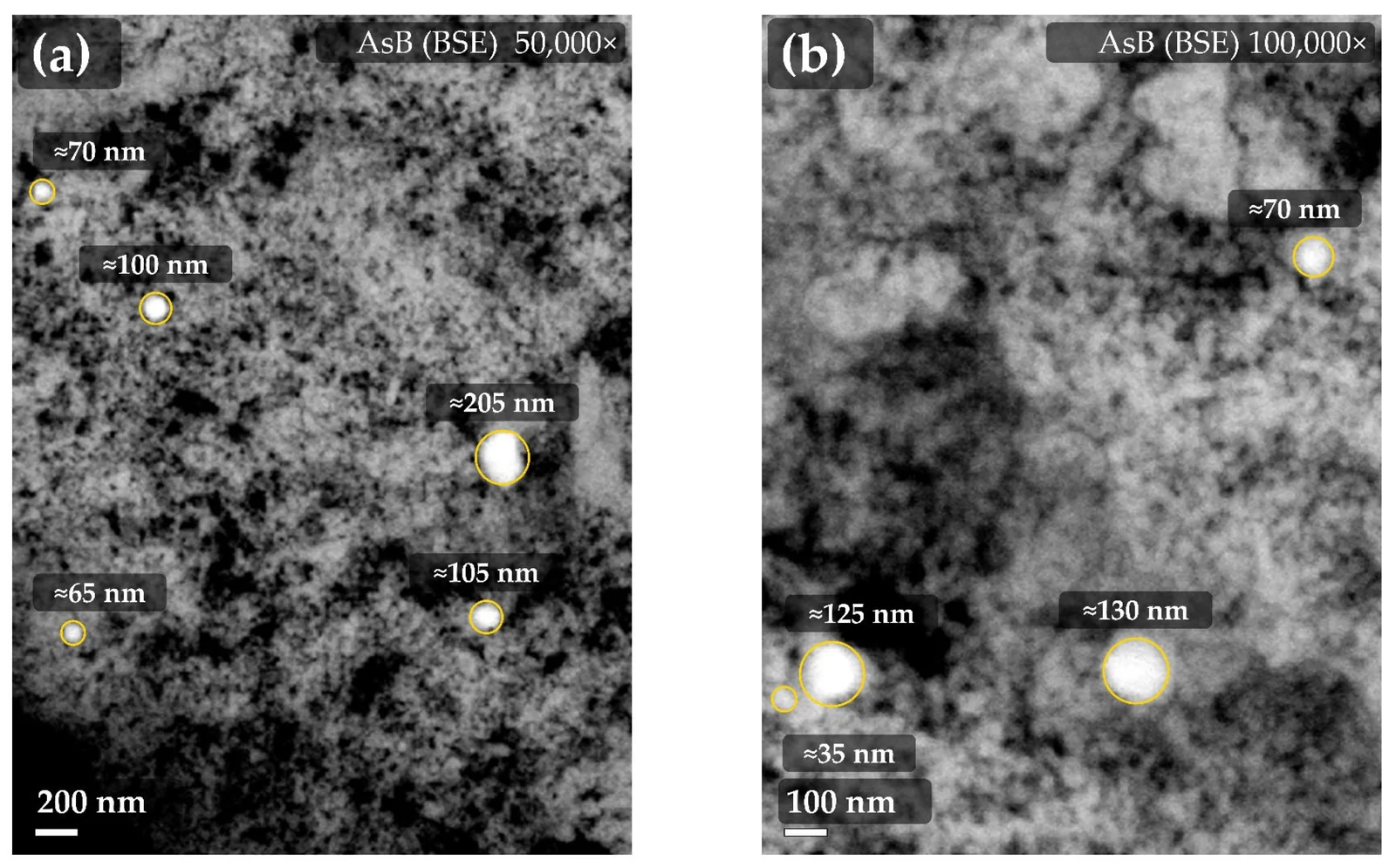
SEM images in compositional contrast (angle-selective backscattered-electron detector, AsB) of the ZnO+2%Ag-1.5%H_2_O nanocomposite obtained by MSS: (**a**) magnification 50,000×, (**b**) magnification 100,000×. The bright features are high-Z objects consistent with an Ag-rich phase. AsB imaging was performed for this sample only.

The AsB image does not identify the element. The only element heavier than zinc detected by EDS is silver (Z = 47 versus 30 for Zn), and therefore the bright objects in Figure 8 are consistent with an Ag-rich phase.

AsB imaging was performed only for the ZnO+2%Ag-1.5%H_2_O sample, at magnifications of 50,000× and 100,000×. In this mode the image brightness depends on the local composition and the mean atomic number, so objects consistent with an Ag-rich phase (Ag, Z = 47), which backscatters electrons more strongly than the ZnO matrix, appear brighter. Several discrete, approximately spherical high-Z features, about 30–210 nm in apparent diameter and most of them in the range 50–150 nm, were observed against the nanometric ZnO matrix; their contrast is consistent with an Ag-rich phase. These features are well separated and dispersed in the field of view. The lateral extent of the bright backscattered-electron contrast does not necessarily correspond to the particle diameter, so the dimensions quoted are apparent feature dimensions. The largest apparent features exceeded the Ag^0^ crystallite size determined for this sample (about 41 nm). This difference may result from broadening of the contrast by the interaction volume, from polycrystallinity or from aggregation. The apparent dimensions of these features were generally larger than the 20–50 nm ZnO particle dimensions observed by SEM, although the two quantities are not directly equivalent.

XRD confirms the presence of a separate crystalline Ag^0^ phase, and EDS showed silver in the analysed areas of all four samples. In the AsB images of the ZnO+2%Ag-1.5%H_2_O sample, high-Z objects were visible whose contrast corresponds to a possible Ag-rich phase. EDS mapping or STEM-EDS would make it possible to determine the composition of these objects and the position of Ag relative to ZnO, and, at sufficient resolution, also the spatial contact between the two phases. Distinguishing the chemical state and structural form of silver would require other methods, e.g., XPS for the chemical state of the surface and HRTEM with electron diffraction for phase identification. The present results therefore do not exclude small amounts of lattice-incorporated, amorphous or surface-oxidised Ag. AsB imaging was performed for only one sample. The similar EDS estimates and the similar morphology in the SEM images are not sufficient to conclude that the silver distribution was the same in all four samples. Differences in the content or distribution of Ag may have affected the sizes obtained in the individual composites, as discussed after Table 5.

The specific surface area decreased over the studied water content range from 50.7 to 29.0 m^2^/g, by about 43%. This is a parameter relevant to processes occurring at the ZnO surface. The sample obtained at the lowest water content has, for the same mass, a surface area about 75% larger than the sample obtained at the highest water content. The release of Zn^2+^ and the formation of reactive oxygen species take place at the oxide surface. Both processes have been linked to the particle size and the specific surface area of ZnO [6,7,35]. The efficiency of photocatalysis also depends on the accessible surface area [10,11]. For ZnO obtained by MSS at different water contents, however, the rate of photocatalytic degradation of phenol increased with particle size [57]. ZnO obtained by MSS (38.8 m^2^/g) and submicron ZnO (4.5 m^2^/g) had a similar effect on the in vitro germination of onion seeds. According to the authors, particle size changes only the rate at which equilibrium is established between ZnO and Zn^2+^ ions, while the final concentration of these ions and germination are determined by the solubility of ZnO [61]. A larger specific surface area therefore need not mean higher activity. The precursor water content makes it possible to set the specific surface area of ZnO/Ag, but its optimum value for a given application has to be determined experimentally. The antimicrobial and photocatalytic activity of the materials obtained was not investigated, and it is not known whether it increases with the specific surface area. For ZnO obtained in different alcohols, the highest photocatalytic and antibacterial activity was shown by the sample with the smallest particles. The authors linked the photocatalytic activity to surface defects, and the higher antibacterial activity to the smaller particle size [36]. The activity is also affected by the amount, distribution and oxidation state of Ag and by the Ag–ZnO contact. These factors may have differed between samples.

Interpreting future activity measurements would require nanoscale characterisation of the Ag–ZnO contact, the junction type (ohmic or rectifying) and the Ag surface oxidation state [12,62].

## 4. Conclusions

This study examined whether ZnO crystallite size shows a similar dependence on precursor water content in ZnO/Ag composites and in a matched Ag-free ZnO reference series. The composites contained a separate metallic Ag^0^ phase formed in situ at a nominal Ag content of 2 mol%. Four ZnO/Ag samples obtained at measured precursor water contents of 1.47–3.97 wt% were compared with the reference series, prepared under the same process conditions.

Microwave solvothermal synthesis from zinc and silver acetates in ethylene glycol, without a separately added reducing agent, produced nanocomposites containing crystalline wurtzite ZnO (P6_3_mc) and a separate crystalline metallic Ag phase (cubic, FCC), with no detectable crystalline silver oxides. The detection of metallic Ag is consistent with reduction of Ag^+^ by ethylene glycol under polyol conditions, although the reaction pathway was not examined directly.

Across the four measured precursor water contents (1.47–3.97 wt%), the apparent ZnO crystallite size, corrected for instrumental broadening, increased monotonically from 15 to 25 nm in the a–b basal plane and from 18 to 40 nm along the c axis, and the crystallites retained the anisotropy characteristic of wurtzite (c/(a–b) ≈ 1.2–1.6). The BET-derived equivalent diameter increased in the same way, from 22 to 38 nm, while the specific surface area decreased from 50.7 to 29.0 m^2^/g. At a nominal Ag content of 2 mol%, this four-point series extends earlier limited observations, including a two-point trend visible in earlier data that were not analysed for the effect of water [38].

The Ag-free ZnO reference series showed a similar dependence on water content, and in both series the direction of change was consistent with earlier results for pure ZnO [25,26]. In the pairs of samples with similar water content, the specific surface area, *d*_(BET)_ and the FW1/5/4/5M mean size of the two series differed by at most 7.3%. Over the common water content range (1.47–3.96 wt%), *d*_(BET)_ increased by about 71% in the composites and by 65% in the reference series. The differences between the series were therefore smaller than the changes associated with water content.

Standardless EDS yielded similar area-averaged Ag estimates but did not provide a bulk Ag balance. AsB imaging of one sample showed high-Z features consistent with an Ag-rich phase; the local Ag–ZnO contact remained unresolved. The skeletal density remained slightly below that of bulk ZnO. The *d*_(BET)_ values and the XRD-derived sizes changed in the same way and had similar values. However, this agreement does not establish how many crystallites each particle contains. Determining this would require TEM [27,57].

The series were prepared separately, and each condition was represented by one synthesis. The comparison is therefore descriptive and cannot isolate the effect of Ag or demonstrate the equivalence of the two systems. The variability between syntheses remains unknown. Assessing it requires independent repeat syntheses, and describing the contribution of Ag requires quantitative determination of the bulk Ag content and TEM/STEM-EDS analysis.

## Figures and Tables

**Table 1:** Summary of the water content analysis of the precursors. The table gives the mean of the determinations together with the standard deviation. In both series, each value is the mean of three determinations.

Sample	Nominal (Target) H_2_O Content [wt%]	Measured H_2_O Content [wt%]
ZnO+2%Ag-1.5%H_2_O	1.5	1.47 ± 0.02
ZnO+2%Ag-2%H_2_O	2	2.01 ± 0.03
ZnO+2%Ag-3%H_2_O	3	3.04 ± 0.03
ZnO+2%Ag-4%H_2_O	4	3.97 ± 0.04
ZnO-1%H_2_O	1	1.12 ± 0.02
ZnO-2%H_2_O	2	1.99 ± 0.03
ZnO-3%H_2_O	3	2.97 ± 0.02
ZnO-4%H_2_O	4	3.96 ± 0.04

**Table 2:** Apparent crystallite sizes determined by XRD (Scherrer equation and FW1/5/4/5M method) and BET-derived equivalent particle diameters of the ZnO/Ag nanocomposites (Section 2.4 and Section 2.5). The Scherrer sizes were determined from the integral breadth at K = 1, after correction for instrumental broadening. The values before correction are given in Table S6 in the Supplementary Materials.

Sample	Measured H_2_O [wt%]	*d*_(BET)_ [nm]	ZnO (100), a–b [nm]	ZnO (002), c [nm]	Ag^0^ (111) [nm]	FW1/5/4/5M, d ± σ [nm]
ZnO+2%Ag-1.5%H_2_O	1.47	22.3	14.7	17.8	41.4	23.2 ± 5.2
ZnO+2%Ag-2%H_2_O	2.01	28.9	18.6	27.1	41.0	29.4 ± 6.2
ZnO+2%Ag-3%H_2_O	3.04	34.9	22.7	33.3	38.7	34.4 ± 8.1
ZnO+2%Ag-4%H_2_O	3.97	38.1	24.9	39.6	40.3	38.7 ± 10.4

**Table 3:** Specific surface area (BET) and skeletal density of ZnO/Ag nanocomposites.

Sample	Measured H_2_O [wt%]	BET Specific Surface Area [m^2^/g]	Density [g/cm^3^]
ZnO+2%Ag-1.5%H_2_O	1.47	50.7	5.318 ± 0.011
ZnO+2%Ag-2%H_2_O	2.01	38.4	5.399 ± 0.009
ZnO+2%Ag-3%H_2_O	3.04	31.8	5.400 ± 0.009
ZnO+2%Ag-4%H_2_O	3.97	29.0	5.428 ± 0.009

**Table 4:** Specific surface area, skeletal density, BET-derived equivalent particle diameter, directional Scherrer sizes and FW1/5/4/5M mean size for the Ag-free ZnO reference series (Section 2.2). The Scherrer sizes were determined from the integral breadth at K = 1, after correction for instrumental broadening. The values obtained before instrumental correction for the Scherrer sizes are given in Table S6 in the Supplementary Materials.

Sample	Measured H_2_O [wt%]	BET Specific Surface Area [m^2^/g]	Density [g/cm^3^]	*d*_(BET)_ [nm]	ZnO (100), a–b [nm]	ZnO (002), c [nm]	FW1/5/4/5M, d ± σ [nm]
ZnO-1%H_2_O	1.12	54.4	5.204 ± 0.012	21.2	13.9	17.4	21.5 ± 4.1
ZnO-2%H_2_O	1.99	37.8	5.273 ± 0.018	30.1	19.6	27.9	31.7 ± 8.2
ZnO-3%H_2_O	2.97	34.2	5.307 ± 0.021	33.1	22.3	29.4	34.2 ± 8.2
ZnO-4%H_2_O	3.96	27.6	5.315 ± 0.020	40.9	25.6	39.9	40.1 ± 11.4

**Table 5:** Average elemental composition of ZnO/Ag nanocomposites obtained by MSS at variable nominal precursor water content (nominally 2 mol% Ag), determined by EDS. For each sample, the normalised concentrations of O, Zn and Ag (sum = 100%) are given as the mean value of three area analyses performed at different locations on the sample ± standard deviation.

Sample	Measured H_2_O [wt%]	Elements					
		O [wt%]	Zn [wt%]	Ag [wt%]	O [at%]	Zn [at%]	Ag [at%]
ZnO+2%Ag-1.5%H_2_O	1.47	19.5 ± 0.3	78.4 ± 0.3	2.16 ± 0.05	49.9 ± 0.4	49.2 ± 0.4	0.82 ± 0.02
ZnO+2%Ag-2%H_2_O	2.01	18.9 ± 0.5	78.9 ± 0.4	2.15 ± 0.20	49.1 ± 0.7	50.1 ± 0.7	0.83 ± 0.08
ZnO+2%Ag-3%H_2_O	3.04	18.6 ± 0.4	79.3 ± 0.5	2.12 ± 0.20	48.5 ± 0.6	50.7 ± 0.7	0.82 ± 0.07
ZnO+2%Ag-4%H_2_O	3.97	18.4 ± 0.3	79.4 ± 0.2	2.20 ± 0.11	48.2 ± 0.4	50.9 ± 0.4	0.86 ± 0.05

## Data Availability

The raw data supporting the conclusions of this article will be made available by the author on request.

## Supplementary Materials

The following supporting information can be downloaded at https://www.mdpi.com/article/10.3390/ma19214520/s1, Figure S1: Diffraction patterns of the Ag-free ZnO reference series; Figure S2: SEM images of the Ag-free ZnO reference series; Figure S3: FW1/5/4/5M crystallite size distributions of the Ag-free ZnO reference series; Table S1: Characterisation of the Ag-free ZnO reference series; Table S2: Records of the FW1/5/4/5M analyses for both series; Table S3: Values used for the comparison in Section 3.3; Table S4: LaB_6_ reflections used for the instrumental correction; Table S5: Regression parameters of the instrumental widths; Table S6: Scherrer sizes before and after instrumental correction; Table S7: Stages of processing the crystallite sizes from the XRD data; Table S8: Anisotropy ratio c/(a–b) of the ZnO crystallites in both series. References [39,41,42,43,44,45] are cited in the Supplementary Materials.

## Abbreviations

The following abbreviations are used in this manuscript:

MSS	Microwave solvothermal synthesis
XRD	X-ray diffraction
BET	Brunauer–Emmett–Teller (method)
SSA	Specific surface area
SEM	Scanning electron microscopy
EDS	Energy-dispersive X-ray spectroscopy
InLens	Immersion-lens secondary-electron detector
SE2	Chamber secondary-electron detector
AsB	Angle-selective backscattered-electron detector
EG	Ethylene glycol
ROS	Reactive oxygen species
FCC	Face-centred cubic
