# Supplementary material for: Water-Content Dependence of ZnO Crystallite Size in Single-Step Microwave Solvothermal Co-Synthesis of ZnO/Ag Nanocomposites
Source: Materials (Basel). 2026 Oct 27;19(21):4520. doi: 10.3390/ma19214520 (PMC13763685; doi:10.3390/ma19214520)
Supplement: Supplementary file 1 [file materials-19-04520-s001.zip › materials-4619583-supplementary.pdf]

Supplementary Materials

Water-Content Dependence of ZnO Crystallite Size in Single-Step Microwave Solvothermal Co-Synthesis of ZnO/Ag Nanocomposites

Jacek Wojnarowicz, Urszula Szałaj and Svitlana Stelmakh

Ag-free ZnO reference series

The reference series was obtained on 3 January 2017 in an ERTEC 02-02 microwave reactor under the same process conditions as the ZnO/Ag series (220 °C, 25 min, nominal power 600 W, 75 mL charge), from the same batches of zinc acetate dihydrate and ethylene glycol, at a zinc concentration of 0.304 mol/L and without silver(I) acetate (Section 2.2 of the main text). All measurements were performed as described in Sections 2.3 to 2.6 of the main text.

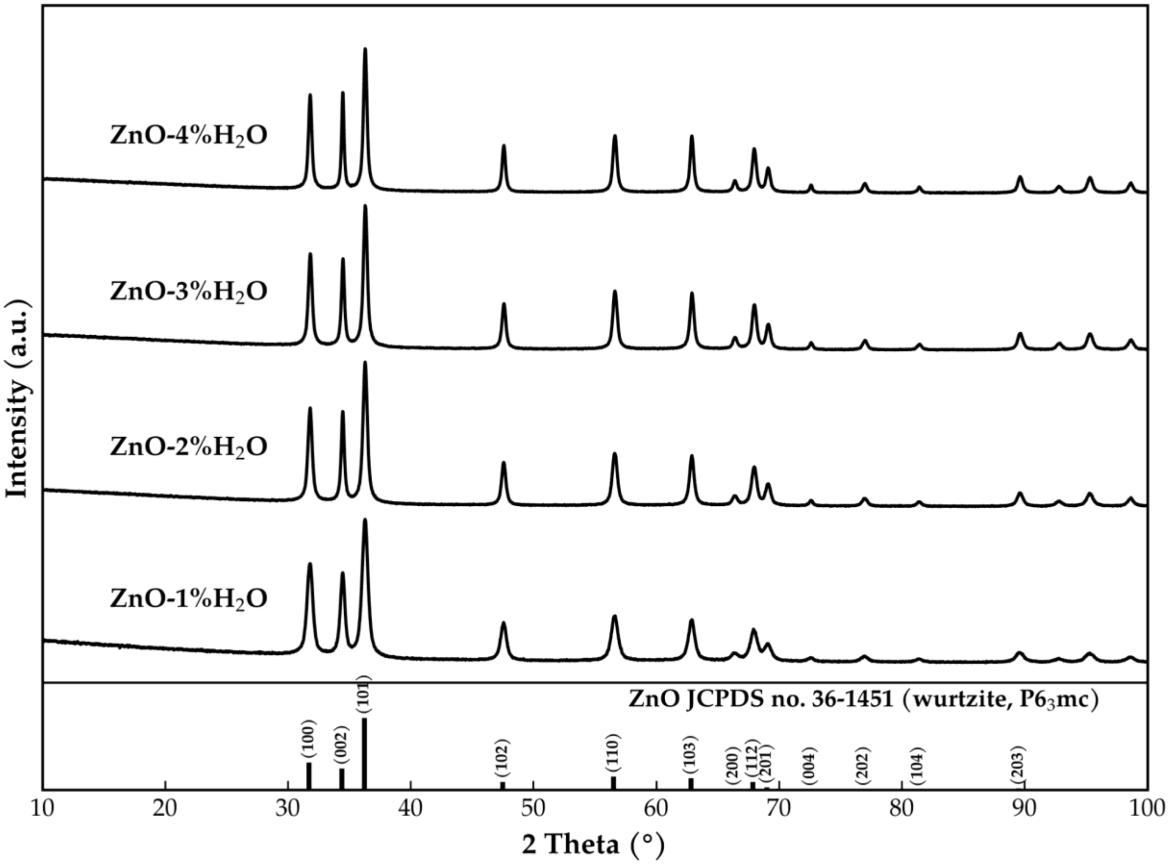

Figure S1. Diffraction patterns of the Ag-free ZnO reference series. Patterns were recorded at nominal precursor water contents of 1, 2, 3 and 4 wt% using $CuK\alpha_1$ radiation with an incident-beam monochromator (λ = 1.54056 Å; 2θ = 10–100°; step 0.03°). The reference pattern of wurtzite ZnO (JCPDS no. 36-1451) is shown at the bottom. All reflections correspond to wurtzite ZnO ($P6_3mc$).

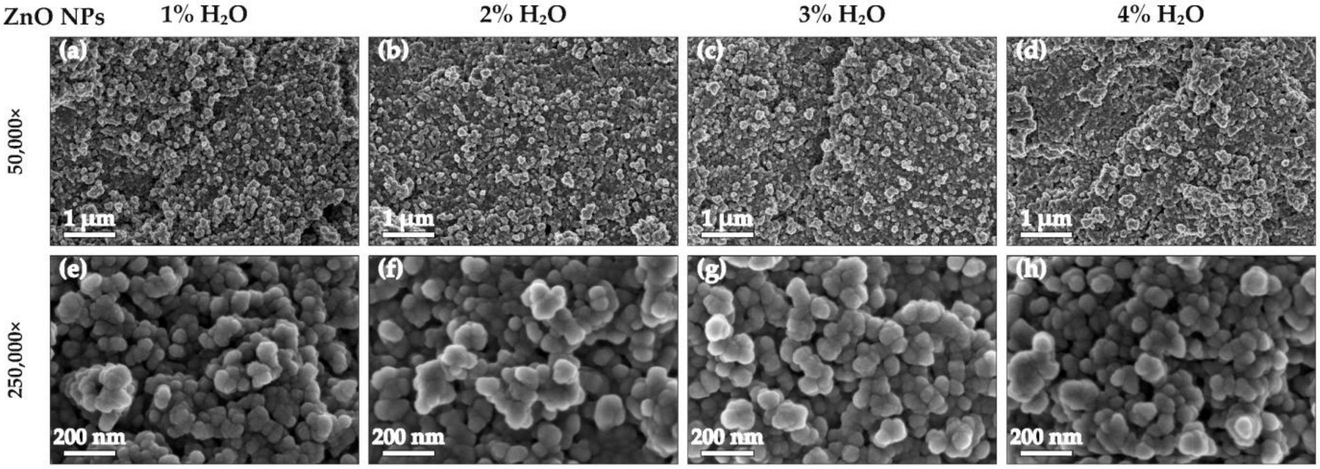

Figure S2. SEM images of the Ag-free ZnO reference series. Columns correspond to nominal precursor water contents of 1, 2, 3 and 4 wt%, from left to right. Magnifications: (a–d) 50,000×; (e–h) 250,000×. Images were acquired with an ULTRA PLUS microscope (ZEISS) using an InLens secondary-electron detector at an accelerating voltage of 2 kV.

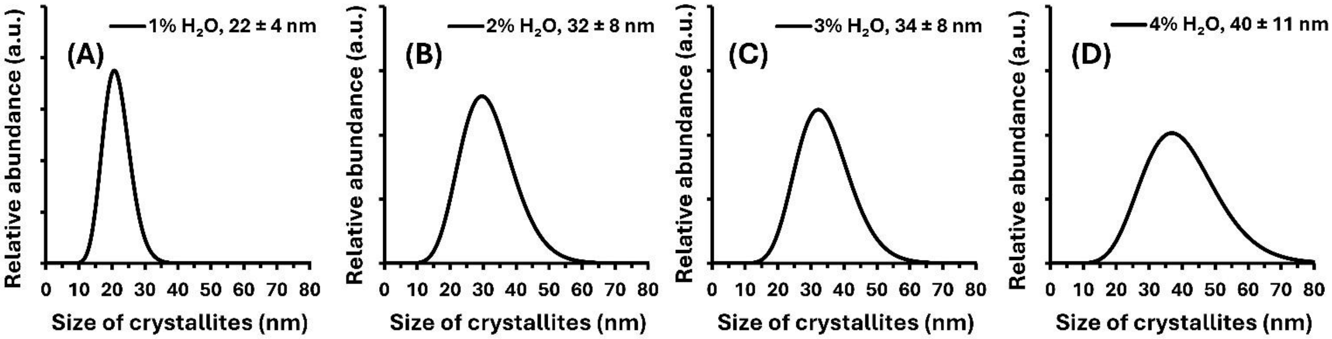

Figure S3. FW1/5/4/5M crystallite size distributions of the Ag-free ZnO reference series. The distributions were determined from XRD line profiles using the FW1/5/4/5M method (Nanopowder XRD Processor Demo, pre-α version 0.0.8, © Pielaszek Research) [43–45] (reference numbers as in the main text). Nominal precursor water contents: (A) 1 wt%, (B) 2 wt%, (C) 3 wt% and (D) 4 wt%.

Table S1. Characterisation of the Ag-free ZnO reference series. Water content: mean ± standard deviation of three determinations by Karl Fischer titration. The specific surface area (SSA) was determined by the BET (Brunauer–Emmett–Teller) method once for each sample; skeletal density (ϱ): mean ± standard deviation of repeated measurement cycles on one specimen. $d_{(BET)} = 6000/(\varrho\cdot SSA)$: equivalent particle diameter in nm (ϱ in g/cm^3, SSA in m^2/g). Scherrer sizes: integral breadth, K = 1, corrected for instrumental broadening using the LaB_6 standard (Tables S4–S6). FW1/5/4/5M: mean size ± width σ of the modelled distribution (not the uncertainty of the mean).

Sample	Nominal H_2O [wt%]	Measured H_2O [wt%]	SSA [m²/g]	Density [g/cm³]	$d_{(BET)}$ [nm]	ZnO (100), a–b [nm]	ZnO (002), c [nm]	c/(a–b)	FW1/5/4/5M, d ± σ [nm]
ZnO-1%H_2O	1	1.12 ± 0.02	54.4	5.204 ± 0.012	21.2	13.9	17.4	1.25	21.5 ± 4.1
ZnO-2%H_2O	2	1.99 ± 0.03	37.8	5.273 ± 0.018	30.1	19.6	27.9	1.42	31.7 ± 8.2
ZnO-3%H_2O	3	2.97 ± 0.02	34.2	5.307 ± 0.021	33.1	22.3	29.4	1.32	34.2 ± 8.2
ZnO-4%H_2O	4	3.96 ± 0.04	27.6	5.315 ± 0.020	40.9	25.6	39.9	1.56	40.1 ± 11.4

Table S2. Records of the FW1/5/4/5M analyses for both series (Nanopowder XRD Processor Demo, pre·α ver. 0.0.8, protocol X10.11a). For the ZnO/Ag samples, trimmed data files ending below the Ag (111) reflection at 2θ ≈ 38.2° were analysed. The reference series was analysed with data files trimmed to a comparable window. Values obtained from the full diffraction patterns of the reference series are given below the table. ⟨R⟩: mean crystallite size, denoted d in Table S1 and in Tables 2 and 4 of the main text; σ: width of the modelled size distribution.

Sample	Data window 2θ [°]	⟨R⟩ [nm]	σ [nm]	σ/⟨R⟩
ZnO-1%H_2O	29.01–38.01	21.5	4.1	0.19
ZnO-2%H_2O	29.01–38.01	31.7	8.2	0.26
ZnO-3%H_2O	29.01–38.01	34.2	8.2	0.24
ZnO-4%H_2O	28.26–37.92	40.1	11.4	0.28
ZnO+2%Ag-1.5%H_2O	28.89–37.74	23.2	5.2	0.22
ZnO+2%Ag-2%H_2O	30.21–37.59	29.4	6.2	0.21
ZnO+2%Ag-3%H_2O	29.73–37.68	34.4	8.1	0.24
ZnO+2%Ag-4%H_2O	29.64–37.59	38.7	10.4	0.27

Sensitivity to the analysed window. For the full diffraction patterns of the reference series, the program used the range 2θ ≈ 25.5–46.5° (scattering vector $q = 4\pi\cdot\sin\theta/\lambda \approx 1.8–3.2$ Å^{-1}). The mean sizes ⟨R⟩ were then 21.4, 31.3, 33.4 and 40.6 nm, and the widths σ were 6.6, 10.6, 11.0 and 14.1 nm (σ/⟨R⟩ = 0.31, 0.34, 0.33 and 0.35) for samples ZnO-1%H_2O to ZnO-4%H_2O. Trimming the window changed ⟨R⟩ by −1.2 to +2.4% and σ by −19 to −38%. Changing the window had little effect on the mean size, but clearly changed the width σ.

Table S3. Values used for the comparison in Section 3.3, calculated from the data given in Tables 1 to 4 of the main text. The comparison method is described in Section 2.7 of the main text. The Scherrer sizes are corrected for instrumental broadening. Slope differences were calculated from unrounded slopes. The slopes b(ZnO) and b(ZnO/Ag) were determined separately for each series and each parameter. A straight line $y = a + b\cdot x$ was fitted to the four points of a series (x_i, y_i), i = 1…4, by ordinary least squares (linear regression), and the slope was calculated from the formula $b = \Sigma(x_i - \bar{x})(y_i - \bar{y}) / \Sigma(x_i - \bar{x})^2$. x_i is the precursor water content measured by Karl Fischer titration [wt%] (Table 1 of the main text): 1.12, 1.99, 2.97 and 3.96 wt% for the ZnO series and 1.47, 2.01, 3.04 and 3.97 wt% for the ZnO/Ag series. y_i is the parameter value for the same sample (Tables 2, 3 and 4 of the main text). For each series, $\bar{x}$ and $\bar{y}$ are the arithmetic means of the four x_i values and the four y_i values, respectively. All

points were weighted equally. The slope gives the change in the parameter for an increase in precursor water content of 1 wt%. The calculations were performed with the SLOPE function in Excel, which implements the same formula. Slope difference: 100 × |b(ZnO/Ag) − b(ZnO)| / |b(ZnO)|. Sample-pair differences: 100 × (ZnO/Ag − ZnO)/ZnO for the pairs of samples at about 2, 3 and 4 wt% H_2O. Deviations: each ZnO/Ag value relative to the reference value at the same water content, obtained by piecewise-linear interpolation of the reference series (linear extrapolation from the last two reference points to 3.97 wt%). RMS: square root of the mean of the squares of the four deviations. These quantities describe differences between individual samples. They are neither measurement uncertainties nor estimates of synthesis variability.

**Parameter**	**Slope, ZnO**	**Slope, ZnO/Ag**	**Slope difference [%]**	**Sample-pair differences [%] (~2, 3, 4 wt%)**	**Deviations [%] (1.47; 2.01; 3.04; 3.97 wt%)**	**RMS [%]**
SSA [m^2/g per wt%]	-8.75	-8.01	8.4	+1.6; -7.0; +5.1	+6.2; +1.8; -5.7; +5.3	5.1
$d_{(BET)}$ [nm per wt%]	+6.51	+6.09	6.4	-4.0; +5.4; -6.8	-10.0; -4.2; +3.7; -7.0	6.7
FW1/5/4/5M, mean [nm per wt%]	+6.09	+5.89	3.2	-7.3; +0.6; -3.5	-9.4; -7.4; -0.6; -3.6	6.3
Scherrer a–b [nm per wt%]	+3.96	+3.98	0.6	-5.1; +1.8; -2.7	-9.2; -5.4; +0.7; -2.9	5.5
Scherrer c [nm per wt%]	+7.24	+8.16	12.8	-2.9; +13.3; -0.8	-17.7; -3.0; +10.5; -1.0	10.4

Sensitivity test. The pair at about 3 wt% H_2O was omitted because the size difference along the c axis in this pair (13.3%) was several times larger than at 2 and 4 wt% (2.9 and 0.8%). Without this pair, the slopes along the c axis are +7.61 (ZnO) and +8.09 nm per wt% (ZnO/Ag), giving a difference of 6.3%; the slopes in the a–b plane are +3.94 and +3.85 nm per wt%, giving a difference of 2.2%. Before instrumental correction (uncorrected pseudo-Voigt profile widths, Table S6), the slopes were +3.52 and +3.49 nm per wt% in the a–b basal plane (difference 0.8%) and +5.85 and +6.48 nm per wt% along the c axis (difference 10.8%).

Correction for instrumental broadening

The Scherrer sizes in the main text were corrected for instrumental broadening using a LaB_6 powder standard. The standard was measured on the same X'Pert PRO diffractometer, after instrument alignment, in the same optical configuration as the samples. This section gives the data and steps needed to repeat the calculations.

The reflections of the samples and of the standard were fitted in Fityk 0.9.8 [39] with one pseudo-Voigt function per reflection. The background was described by a quadratic function for the standard and by a linear function for the samples. The fit gives the peak position 2θ, the half width at half maximum (hwhm), the shape parameter η, the peak area A and the peak height I_0. The subsequent calculations comprise six steps.

Step 1. Integral breadth and shape factor: $\beta = A/I_0$, FWHM = 2·hwhm and φ = FWHM/β.

Step 2. Lorentzian and Gaussian components according to de Keijser et al. [41] (J. Appl. Cryst. 1982, 15, 308–314, equations (3)–(5)): $\beta_L = \beta(2.0207 - 0.4803\varphi - 1.7756\varphi^2)$ and $\beta_G = \beta[0.6420 + 1.4187(\varphi - 2/\pi)^{1/2} - 2.2043\varphi + 1.8706\varphi^2]$.
Step 3. Instrumental components. Steps 1 and 2 were applied to the (110), (111) and (200) reflections of LaB_6 (Table S4). Straight lines $\beta_{L,inst} = a_L \cdot 2\theta + b_L$ and $\beta_{G,inst} = a_G \cdot 2\theta + b_G$ were fitted to these values by least squares (Table S5). All reflections used to calculate the crystallite sizes (2θ = 31.8–38.2°) lie within the range covered by the standard (30.5–43.6°), so the lines were used for interpolation only.
Step 4. Removal of the instrumental contribution according to Langford [42] (J. Appl. Cryst. 1978, 11, 10–14, equations (5) and (11)): $\beta_{L,s} = \beta_L - \beta_{L,inst}$ and $\beta_{G,s} = (\beta_G^2 - \beta_{G,inst}^2)^{1/2}$. The subscript s denotes the components of the sample profile after removal of the instrumental contribution.
Step 5. Integral breadth of the corrected Voigt profile according to Langford [42] (1978, equations (19) and (25)): $k = \beta_{L,s}/(\pi^{1/2} \cdot \beta_{G,s})$ and $\beta_s = \beta_{G,s} \cdot \exp(-k^2)/\mathrm{erfc}(k)$, where erfc denotes the complementary error function.
Step 6. Scherrer equation: $D = K\lambda/(\beta \cdot \cos\theta)$, where the Scherrer constant K = 1, λ = 0.1540562 nm and β is expressed in radians. The size before correction was calculated from β obtained in Step 1, and the size after correction from β_s obtained in Step 5 (Table S6).
The microstrain contribution was not separated from the size contribution, so the corrected sizes remain apparent values.

Table S4. LaB_6 reflections used for the instrumental correction. The reflections were fitted with pseudo-Voigt functions in Fityk 0.9.8. $\beta = A/I_0$; φ = FWHM/β; β_L and β_G were calculated as described in Step 2.

Reflection	2θ [°]	hwhm [°]	η	Area A	Height I_0	FWHM [°]	β [°]	φ	β_L [°]	β_G [°]
(110)	30.461	0.03476	0.142	551.3	6977.5	0.06953	0.07901	0.8799	0.01764	0.06721
(111)	37.518	0.03451	0.222	257.9	3166.6	0.06903	0.08143	0.8477	0.02750	0.06265
(200)	43.585	0.03385	0.306	133.8	1620.8	0.06770	0.08254	0.8201	0.03569	0.05779

Table S5. Regression parameters of the instrumental widths. Straight lines $\beta_{inst} = a \cdot 2\theta + b$ were fitted by least squares (linear regression) to the instrumental components of the three LaB_6 reflections (Table S4). The coefficient a is expressed in degrees per degree 2θ, and b in degrees.

Component	a	b [°]	R^2
$\beta_{L,inst}$	0.00137652	-0.0242481	0.99991
$\beta_{G,inst}$	-0.00071551	0.0891595	0.99613

Table S6. Scherrer sizes before and after instrumental correction for all fitted reflections. FWHM and β: measured pseudo-Voigt profile (Step 1). $\beta_{L,inst}$ and $\beta_{G,inst}$: instrumental components at the 2θ value of the given reflection (Table S5). β_s: corrected integral breadth of the Voigt profile (Step 5). D: apparent volume-weighted size (K = 1, λ = 0.1540562 nm).

Change: 100 × (D after correction/D before correction − 1). Only the values for (100), (002) and Ag (111) are used in Tables 2 and 4 of the main text.

Sample	Reflection	2θ [°]	FWHM [°]	β [°]	$\beta_{L,inst}$ [°]	$\beta_{G,inst}$ [°]	β_s [°]	D before correction [nm]	D after correction [nm]	Change [%]
ZnO+2%Ag-1.5%H_2O	ZnO (100)	31.837	0.48140	0.64390	0.01958	0.06638	0.62267	14.3	14.7	+3.4
ZnO+2%Ag-1.5%H_2O	ZnO (002)	34.501	0.39206	0.54387	0.02324	0.06447	0.51826	17.0	17.8	+4.9
ZnO+2%Ag-1.5%H_2O	ZnO (101)	36.319	0.48782	0.65313	0.02575	0.06317	0.62762	14.2	14.8	+4.1
ZnO+2%Ag-1.5%H_2O	Ag (111)	38.196	0.18568	0.26718	0.02833	0.06183	0.22578	35.0	41.4	+18.3
ZnO+2%Ag-2%H_2O	ZnO (100)	31.835	0.38548	0.51758	0.01957	0.06638	0.49466	17.7	18.6	+4.6
ZnO+2%Ag-2%H_2O	ZnO (002)	34.500	0.26278	0.37201	0.02324	0.06447	0.34110	24.8	27.1	+9.1
ZnO+2%Ag-2%H_2O	ZnO (101)	36.320	0.38945	0.51734	0.02575	0.06317	0.49034	18.0	18.9	+5.5
ZnO+2%Ag-2%H_2O	Ag (111)	38.190	0.18392	0.27303	0.02832	0.06183	0.22800	34.2	41.0	+19.8
ZnO+2%Ag-3%H_2O	ZnO (100)	31.832	0.32128	0.42814	0.01957	0.06638	0.40359	21.4	22.7	+6.1
ZnO+2%Ag-3%H_2O	ZnO (002)	34.495	0.22554	0.30950	0.02323	0.06448	0.27752	29.9	33.3	+11.5
ZnO+2%Ag-3%H_2O	ZnO (101)	36.317	0.32307	0.42993	0.02574	0.06317	0.40141	21.6	23.1	+7.1
ZnO+2%Ag-3%H_2O	Ag (111)	38.183	0.19190	0.28613	0.02831	0.06184	0.24162	32.6	38.7	+18.4
ZnO+2%Ag-4%H_2O	ZnO (100)	31.830	0.28888	0.39515	0.01957	0.06638	0.36902	23.2	24.9	+7.1
ZnO+2%Ag-4%H_2O	ZnO (002)	34.492	0.19247	0.26933	0.02323	0.06448	0.23357	34.3	39.6	+15.3
ZnO+2%Ag-4%H_2O	ZnO (101)	36.315	0.29665	0.39743	0.02574	0.06318	0.36800	23.4	25.2	+8.0
ZnO+2%Ag-4%H_2O	Ag (111)	38.181	0.18520	0.27907	0.02831	0.06184	0.23189	33.5	40.3	+20.3
ZnO-1%H_2O	ZnO (100)	31.804	0.50635	0.67928	0.01953	0.06640	0.65846	13.5	13.9	+3.2
ZnO-1%H_2O	ZnO (002)	34.467	0.40430	0.55633	0.02320	0.06450	0.53114	16.6	17.4	+4.7
ZnO-1%H_2O	ZnO (101)	36.291	0.51407	0.68427	0.02571	0.06319	0.65908	13.6	14.1	+3.8
ZnO-2%H_2O	ZnO (100)	31.817	0.36462	0.49267	0.01955	0.06639	0.46923	18.6	19.6	+5.0
ZnO-2%H_2O	ZnO (002)	34.479	0.25815	0.36191	0.02321	0.06449	0.33117	25.5	27.9	+9.3
ZnO-2%H_2O	ZnO (101)	36.302	0.37029	0.49569	0.02572	0.06318	0.46827	18.7	19.8	+5.9
ZnO-3%H_2O	ZnO (100)	31.834	0.32378	0.43709	0.01957	0.06638	0.41248	21.0	22.3	+6.0
ZnO-3%H_2O	ZnO (002)	34.494	0.24985	0.34554	0.02323	0.06448	0.31482	26.7	29.4	+9.8
ZnO-3%H_2O	ZnO (101)	36.319	0.32657	0.43770	0.02575	0.06317	0.40918	21.2	22.7	+7.0
ZnO-4%H_2O	ZnO (100)	31.824	0.28490	0.38388	0.01956	0.06639	0.35788	23.9	25.6	+7.3
ZnO-4%H_2O	ZnO (002)	34.483	0.19515	0.26593	0.02322	0.06449	0.23177	34.8	39.9	+14.7
ZnO-4%H_2O	ZnO (101)	36.309	0.29183	0.38900	0.02573	0.06318	0.35951	23.9	25.8	+8.2

Table S7. Stages of processing the crystallite sizes from the XRD data for the reflections used in the main text. Stage 1: values from Table 2 of the original version of the manuscript, calculated from the full width at half maximum (FWHM) and given to the nearest 1 nm. The reference series was not included in the original version, so Stage 1 does not apply to it (–). Stage 2: sizes calculated from the integral breadth of the Pearson VII profile, without correction for instrumental broadening. Stage 3: sizes calculated from the integral breadth of the pseudo-Voigt profile, without correction for instrumental broadening (Table S6). Stage 4: as in Stage 3, but with correction for instrumental broadening (Tables 2 and 4 of the main text). Total change (Stage 2→4): 100 × (Stage 4/Stage 2 − 1), calculated from unrounded sizes.

Sample	Reflection	Stage 1: size calculated from FWHM [nm]	Stage 2: Pearson VII [nm]	Stage 3: pseudo-Voigt [nm]	Stage 4: after correction [nm]	Total change (Stage 2→4) [%]
ZnO+2%Ag-1.5%H_2O	ZnO (100)	14	15.3	14.3	14.7	-3.7
ZnO+2%Ag-1.5%H_2O	ZnO (002)	23	17.2	17.0	17.8	+3.7
ZnO+2%Ag-1.5%H_2O	Ag (111)	63	34.1	35.0	41.4	+21.3
ZnO+2%Ag-2%H_2O	ZnO (100)	19	19.2	17.7	18.6	-3.4

Sample	Reflection	Stage 1: size calculated from FWHM [nm]	Stage 2: Pearson VII [nm]	Stage 3: pseudo-Voigt [nm]	Stage 4: after correction [nm]	Total change (Stage 2→4) [%]
ZnO+2%Ag-2%H_2O	ZnO (002)	30	25.5	24.8	27.1	+6.3
ZnO+2%Ag-2%H_2O	Ag (111)	58	33.8	34.2	41.0	+21.2
ZnO+2%Ag-3%H_2O	ZnO (100)	23	22.9	21.4	22.7	-0.7
ZnO+2%Ag-3%H_2O	ZnO (002)	38	31.0	29.9	33.3	+7.4
ZnO+2%Ag-3%H_2O	Ag (111)	52	32.3	32.6	38.7	+19.7
ZnO+2%Ag-4%H_2O	ZnO (100)	26	25.1	23.2	24.9	-0.9
ZnO+2%Ag-4%H_2O	ZnO (002)	46	35.5	34.3	39.6	+11.5
ZnO+2%Ag-4%H_2O	Ag (111)	52	30.6	33.5	40.3	+31.6
ZnO-1%H_2O	ZnO (100)	–	14.3	13.5	13.9	-2.5
ZnO-1%H_2O	ZnO (002)	–	16.9	16.6	17.4	+3.0
ZnO-2%H_2O	ZnO (100)	–	19.7	18.6	19.6	-0.7
ZnO-2%H_2O	ZnO (002)	–	25.9	25.5	27.9	+7.7
ZnO-3%H_2O	ZnO (100)	–	22.2	21.0	22.3	+0.2
ZnO-3%H_2O	ZnO (002)	–	27.6	26.7	29.4	+6.4
ZnO-4%H_2O	ZnO (100)	–	25.4	23.9	25.6	+1.0
ZnO-4%H_2O	ZnO (002)	–	36.2	34.8	39.9	+10.2

Table S8. Anisotropy ratio c/(a–b) of the ZnO crystallites in both series. The table compares the Ag-free ZnO reference series and the ZnO/Ag composite series. The ratio was calculated as the quotient of the apparent crystallite size along the c axis, determined from the (002) reflection, and the apparent size in the a–b basal plane, determined from the (100) reflection. Both sizes were determined from the Scherrer equation with the integral breadth corrected for instrumental broadening, at K = 1, and were taken from Tables 2 and 4 of the main text. The ratio was calculated from values rounded to 0.1 nm.

Series	Sample	Measured H_2O content [wt%]	ZnO (100), a–b [nm]	ZnO (002), c [nm]	c/(a–b)
ZnO	ZnO-1%H_2O	1.12	13.9	17.4	1.25
ZnO	ZnO-2%H_2O	1.99	19.6	27.9	1.42
ZnO	ZnO-3%H_2O	2.97	22.3	29.4	1.32
ZnO	ZnO-4%H_2O	3.96	25.6	39.9	1.56
ZnO/Ag	ZnO+2%Ag-1.5%H_2O	1.47	14.7	17.8	1.21
ZnO/Ag	ZnO+2%Ag-2%H_2O	2.01	18.6	27.1	1.46
ZnO/Ag	ZnO+2%Ag-3%H_2O	3.04	22.7	33.3	1.47
ZnO/Ag	ZnO+2%Ag-4%H_2O	3.97	24.9	39.6	1.59

References

Reference numbering follows the main manuscript.

[39] Wojdyr, M. Fityk: A general-purpose peak fitting program. J. Appl. Crystallogr. 2010, 43, 1126–1128. https://doi.org/10.1107/S0021889810030499.
[41] de Keijser, T.H.; Langford, J.I.; Mittemeijer, E.J.; Vogels, A.B.P. Use of the Voigt function in a single-line method for the analysis of X-ray diffraction line broadening. J. Appl. Crystallogr. 1982, 15, 308–314. https://doi.org/10.1107/S0021889882012035.

[42] Langford, J.I. A rapid method for analysing the breadths of diffraction and spectral lines using the Voigt function. J. Appl. Crystallogr. 1978, 11, 10–14. https://doi.org/10.1107/S0021889878012601.
[43] Pielaszek, R. FW1/5/4/5M method for determination of the grain size distribution from powder diffraction line profile. J. Alloys Compd. 2004, 382, 128–132. https://doi.org/10.1016/j.jallcom.2004.05.040.
[44] Nanopowder XRD Processor Demo, pre·α·ver.0.0.8, © Pielaszek Research. Available online: http://science24.com/xrd/ (accessed on 1 June 2026).
[45] FW1/5/4/5M Method of Evaluation of Grain Size Distribution by Powder Diffraction. Available online: http://science24.com/fw145m/ (accessed on 1 June 2026).
